# Large Language Models in Summarizing Radiology Report Impressions for Lung Cancer in Chinese: Evaluation Study

**DOI:** 10.2196/65547

**Published:** 2025-04-03

**Authors:** Danqing Hu, Shanyuan Zhang, Qing Liu, Xiaofeng Zhu, Bing Liu

**Affiliations:** 1 Jiangsu Key Laboratory of Intelligent Medical Image Computing School of Future Technology Nanjing University of Information Science and Technology Nanjing China; 2 Zhejiang Lab Hangzhou China; 3 Key Laboratory of Carcinogenesis and Translational Research (Ministry of Education) Department of Thoracic Surgery II Peking University Cancer Hospital and Institute Beijing China; 4 Department of Radiology Peking University Cancer Hospital and Institute Beijing China

**Keywords:** large language model, impression summarization, radiology report, radiology, evaluation study, ChatGPT, natural language processing, ultrasound, radiologist, thoracic surgeons

## Abstract

**Background:**

Large language models (LLMs), such as ChatGPT, have demonstrated impressive capabilities in various natural language processing tasks, particularly in text generation. However, their effectiveness in summarizing radiology report impressions remains uncertain.

**Objective:**

This study aims to evaluate the capability of nine LLMs, that is, Tongyi Qianwen, ERNIE Bot, ChatGPT, Bard, Claude, Baichuan, ChatGLM, HuatuoGPT, and ChatGLM-Med, in summarizing Chinese radiology report impressions for lung cancer.

**Methods:**

We collected 100 Chinese computed tomography (CT), positron emission tomography (PET)–CT, and ultrasound (US) reports each from Peking University Cancer Hospital and Institute. All these reports were from patients with suspected or confirmed lung cancer. Using these reports, we created zero-shot, one-shot, and three-shot prompts with or without complete example reports as inputs to generate impressions. We used both automatic quantitative evaluation metrics and five human evaluation metrics (completeness, correctness, conciseness, verisimilitude, and replaceability) to assess the generated impressions. Two thoracic surgeons (SZ and BL) and one radiologist (QL) compared the generated impressions with reference impressions, scoring them according to the five human evaluation metrics.

**Results:**

In the automatic quantitative evaluation, ERNIE Bot, Tongyi Qianwen, and Claude demonstrated the best overall performance in generating impressions for CT, PET-CT, and US reports, respectively. In the human semantic evaluation, ERNIE Bot outperformed the other LLMs in terms of conciseness, verisimilitude, and replaceability on CT impression generation, while its completeness and correctness scores were comparable to those of other LLMs. Tongyi Qianwen excelled in PET-CT impression generation, with the highest scores for correctness, conciseness, verisimilitude, and replaceability. Claude achieved the best conciseness, verisimilitude, and replaceability scores on US impression generation, and its completeness and correctness scores are close to the best results obtained by other LLMs. The generated impressions were generally complete and correct but lacked conciseness and verisimilitude. Although one-shot and few-shot prompts improved conciseness and verisimilitude, clinicians noted a significant gap between the generated impressions and those written by radiologists.

**Conclusions:**

Current LLMs can produce radiology impressions with high completeness and correctness but fall short in conciseness and verisimilitude, indicating they cannot yet fully replace impressions written by radiologists.

## Introduction

Clinical documentation plays an indispensable role in health care practice, serving as a primary medium for conveying patient information in clinical settings. Clinicians routinely spend a substantial amount of time summarizing vast amounts of textual information, such as compiling diagnostic reports, writing progress notes, or synthesizing a patient’s treatment history [1,2]. With the increasing adoption of electronic medical record systems, the burden of clinical documentation expanded rapidly, contributing to clinician stress and burnout [3]. A recent study indicates that clinicians spend up to 2 hours on documentation for every hour of patient interaction [4]. This issue may be more prominent in countries with large populations and limited health care resources.

As one of the most important clinical documents, radiology reports record essential information from patient imaging data such as computed tomography (CT) scans, positron emission tomography (PET) scans, magnetic resonance imaging, x-rays, and ultrasound (US) examinations. These reports typically consist of two main sections, namely findings and impressions. The findings section presents the radiologist’s observations from the images, while the impressions section provides a concise summary of the observed abnormalities and corresponding diagnoses or suspicions with tendencies. Radiology reports, especially the impressions, are crucial for patient diagnosis, disease progression assessment, and treatment planning. Impression summarization refers to using models to simulate physicians to condense lengthy and detailed findings into concise and informative impressions [5-7], which is a key application of text summarization in the medical field [8]. This technique may greatly relieve the workload of radiologists and reduce their possible errors and omissions, thereby improving the accuracy of clinical evaluations [9-11].

Recently, large language models (LLMs) such as ChatGPT [12] and GPT-4 [13] have captured worldwide attention due to their astonishing text-generation capabilities. Through pretraining on vast amounts of data, LLMs demonstrate remarkable performance on unseen downstream tasks using zero-shot, one-shot, or few-shot prompts without parameter updates [14]. By reinforcement learning from human feedback (RLHF) [15], the LLMs are further guaranteed to produce harmless and unbiased content that aligns with human expectations. The great success of prompt-based LLMs has led to a paradigm shift in natural language processing research [16-21], thereby bringing new opportunities for radiology report impression summarization.

To enable LLMs to effectively perform specific tasks like impression summarization, it is essential to craft instructions that LLMs can accurately interpret and execute, a process known as prompt engineering. Few-shot prompting is one of the most effective prompt engineering techniques, which supplies the LLMs with some examples to refine their responses. When applied to impression summarization, few-shot prompting can help the LLMs capture the critical information radiologists prioritize and the writing style they use, which is crucial for generating more realistic impressions. Although some studies have applied prompt-based LLMs to this task [2,22,23], they only focus on limited types of reports, typically the x-ray reports, and lack detailed clinical expert evaluation of the generated results [22,23] or only evaluate the LLMs on English reports in a zero-shot manner [2].

In this study, we conducted a systematic study to explore the capability of prompt-based LLMs in summarizing the impressions of various types of Chinese radiology reports using zero-shot and few-shot prompts. By leveraging automatic quantitative and clinical expert evaluations, we aim to clarify the current status of LLMs in Chinese radiology report impression summarization and the gap between the current achievements and requirements for application in clinical practice.

## Methods

### Study Overview

**Figure 1:**
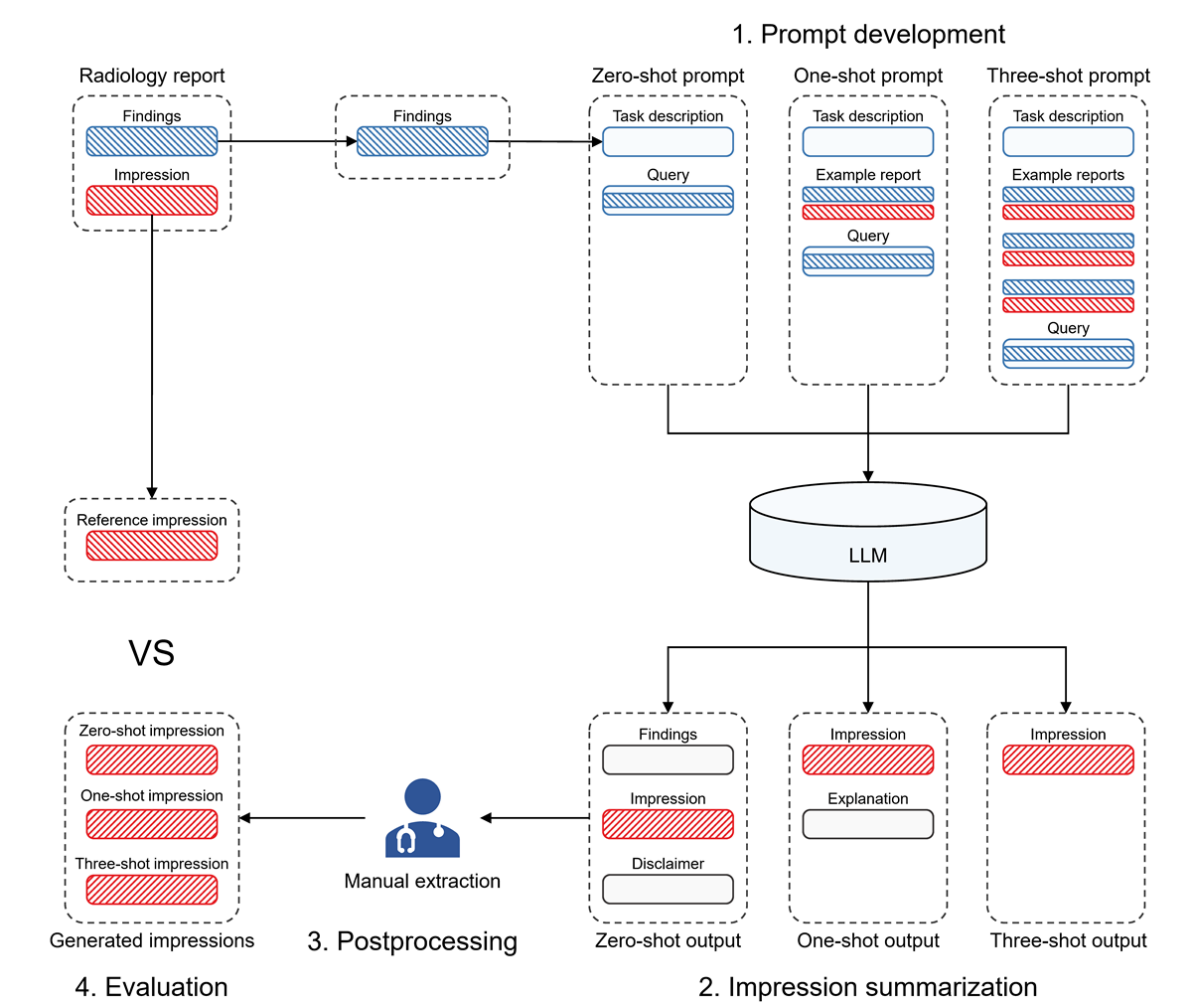
Study overview: (1) Prompt development: we first developed the zero-shot, one-shot, and three-shot prompts for the collected radiology reports using the prompt templates. (2) Impression summarization: we input the prompts into LLMs and collected the outputs with summarized impressions. (3) Postprocessing: we manually extracted the impression-related contents from the LLMs’ outputs. (4) Evaluation: we conducted automatic and human evaluations of the generated impressions. LLM: large language model.

To evaluate the LLMs for impression summarization, we first collected three types of Chinese radiology reports, that is, PET-CT, CT, and US reports from Peking University Cancer Hospital and Institute. Using the collected reports, we evaluated the zero-shot, one-shot, and three-shot performance of impression summarization of five commercially available LLMs, including Tongyi Qianwen, ERNIE Bot, ChatGPT, Bard, and Claude, and four open-source LLMs, including Baichuan, ChatGLM, HuatuoGPT, and ChatGLM-Med. Since the LLMs’ output not only contains the generated impression but also contains some content unrelated to the impression, such as some explanations about how they generate the impressions or disclaimers indicating the generated impressions are for reference only, we manually extracted the impression-related content from the outputs for the automatic quantitative and human sematic evaluations. The overall pipeline is shown in Figure 1.

### Materials

We collected three types of radiology reports, that is, whole body PET-CT, chest CT, and neck and abdomen US reports from Peking University Cancer Hospital and Institute. The relevant patients are all outpatients and inpatients of the Department of Thoracic Surgery II with suspected or confirmed lung cancer. After removing the incomplete reports, we finally obtained 867 PET-CT reports, 819 CT reports, and 1487 US reports. We randomly selected 100 reports from each type of report for automatic quantitative and human evaluations. We manually reviewed the selected reports to make sure that no patient identification information was recorded in these reports to protect patient privacy.

### LLMs

#### Overview

To conduct a comprehensive evaluation, we selected five commercially available and four open-source LLMs with different architectures and parameter sizes. The introduction of the selected LLMs is listed below. Table 1 shows the summary of those LLMs.

**Table 1:** The detailed information on the LLMs^a^ used in this study.

Model	Developer	Type	License	Language support	Maximum input character limits	Version and Last access date
Tongyi Qianwen	Alibaba Cloud	General	Commercially available	Chinese, English	10,000 characters^b^	Tongyi Qianwen v2.1.1/ January 11, 2024
ERNIE Bot	Baidu	General	Commercially available	Chinese, English	2000 characters^b^	ERNIE Bot v2.5.2/ January 11, 2024
ChatGPT	OpenAI	General	Commercially available	Chinese, English	8192 tokens^b^	GPT-3.5/January 11, 2024
Bard	Google	General	Commercially available	Chinese, English	32,000 characters^b^	Bard/January 12, 2024
Claude	Anthropic	General	Commercially available	Chinese, English	200,000 characters	Claude 3.5 Sonnet/October 12, 2024
Baichuan	Baichuan Intelligence	General	Open-source	Chinese, English	4096 tokens	Baichuan-13B-Chat/ January 19, 2024
ChatGLM	Tsinghua University	General	Open-source	Chinese, English	8192 tokens	ChatGLM3-6B/ January 19, 2024
HuatuoGPT	Shenzhen Research Institute of Big Data	Medical	Open-source	Chinese, English	4096 tokens	HuatuoGPT-7B/ January 19, 2024
ChatGLM-Med	Harbin Institution of Technology	Medical	Open-source	Chinese, English	2048 tokens	ChatGLM-Med/ January 19, 2024

^a^LLM: large language model.

^b^The maximum length of text that can be entered on the web interface.

#### Tongyi Qianwen

Tongyi Qianwen is an LLM chat product developed by Alibaba Cloud. The latest Tongyi Qianwen 2.0 extends the Qwen model [24] to a few hundred billion parameters, achieving a substantial upgrade from its predecessor in understanding complex instructions, reasoning, memorizing, and preventing hallucinations. We used Tongyi Qianwen v2.1.1 [25] to generate the impressions.

#### ERNIE Bot

ERNIE Bot (Wenxin Yiyan) is an LLM chat product developed by Baidu based on their ERNIE (Enhanced Representation through Knowledge Integration) [26] and PLATO (Pretrained Dialogue Generation Model) [27] models. Based on the supervised fine-tuning, RLHF, and knowledge, search, and dialogue enhancements, the ERNIE Bot achieves a more precise understanding of the Chinese language and its practical applications. We used ERNIE Bot v2.5.2 [28] to generate the impressions.

#### ChatGPT

ChatGPT is the most impactful LLM developed by OpenAI, raising the trend of prompt-based LLMs worldwide. ChatGPT is an advanced version of instructionGPT [15], which first fine-tunes GPT-3 [14] using human-written demonstrations of the desired output to prompts and then further fine-tuning the model through the RLHF strategy to align language models with user intent. We accessed the ChatGPT via the website interface [29] to obtain the generated impressions before January 11, 2024.

#### Bard

Bard is an LLM chat product powered by Pathways Language Model 2 [30] developed by Google AI. Pathways Language Model 2 is a transformer-based model trained using a mixture of objectives and multilingual datasets, achieving better performances on natural language generation, code generation, translation, and reasoning than its predecessor, PaLM [31]. We accessed the Bard via the website interface [32] to obtain the generated impressions before January 12, 2024.

#### Claude

Claude is a series of LLM chat products developed by Anthropic. Claude models are transformers pretrained to predict the next word in large amounts of text. They are fine-tuned with Constitutional AI [33] to make them harmless and helpful without relying on extensive human feedback. In this study, we accessed the latest Claude 3.5 Sonnet via the website interface [34] before October 12, 2024, to produce the impressions.

#### Baichuan

Baichuan-13B is an open-source LLM developed by Baichuan Intelligence. The Baichuan-13B model has 130 billion parameters trained on 1.4 trillion tokens. It supports both Chinese and English and achieves competitive performance in standard Chinese and English benchmarks among models of its size. We used the Baichuan-13B-Chat [35] to generate the impressions.

#### ChatGLM

ChatGLM3-6B is the latest open-source model in the ChatGLM [36] series developed by Tsinghua University. The ChatGLM3-6B has a more powerful base model trained on a more diverse dataset, sufficient training steps, and a more reasonable training strategy, showing strong performance on language understanding, reasoning, coding, etc. We used the ChatGLM3-6b [37] to generate the impressions.

#### HuatuoGPT

HuatuoGPT [38] is an open-source LLM developed by the Shenzhen Research Institute of Big Data. HuatuoGPT-7B first uses the Baichuan-7B as the backbone model and then uses the distilled data from ChatGPT and real-world data from doctors to supervised fine-tuning and reinforcement learning with mixed feedback to achieve state-of-the-art results in performing medical consultation. We used the HuatuoGPT-7B [39] to generate the impressions.

#### ChatGLM-Med

ChatGLM-Med is an open-source LLM developed by the Harbin Institution of Technology. The ChatGLM-Med uses the ChatGLM-6B as the base model and fine-tunes on a Chinese medical instruction dataset developed by a medical knowledge graph and GPT-3.5 to improve better question-answering results in the medical field. We used the ChatGLM-Med [40] to generate the impressions.

### Impression Summarization Using LLMs

**Figure 2:**
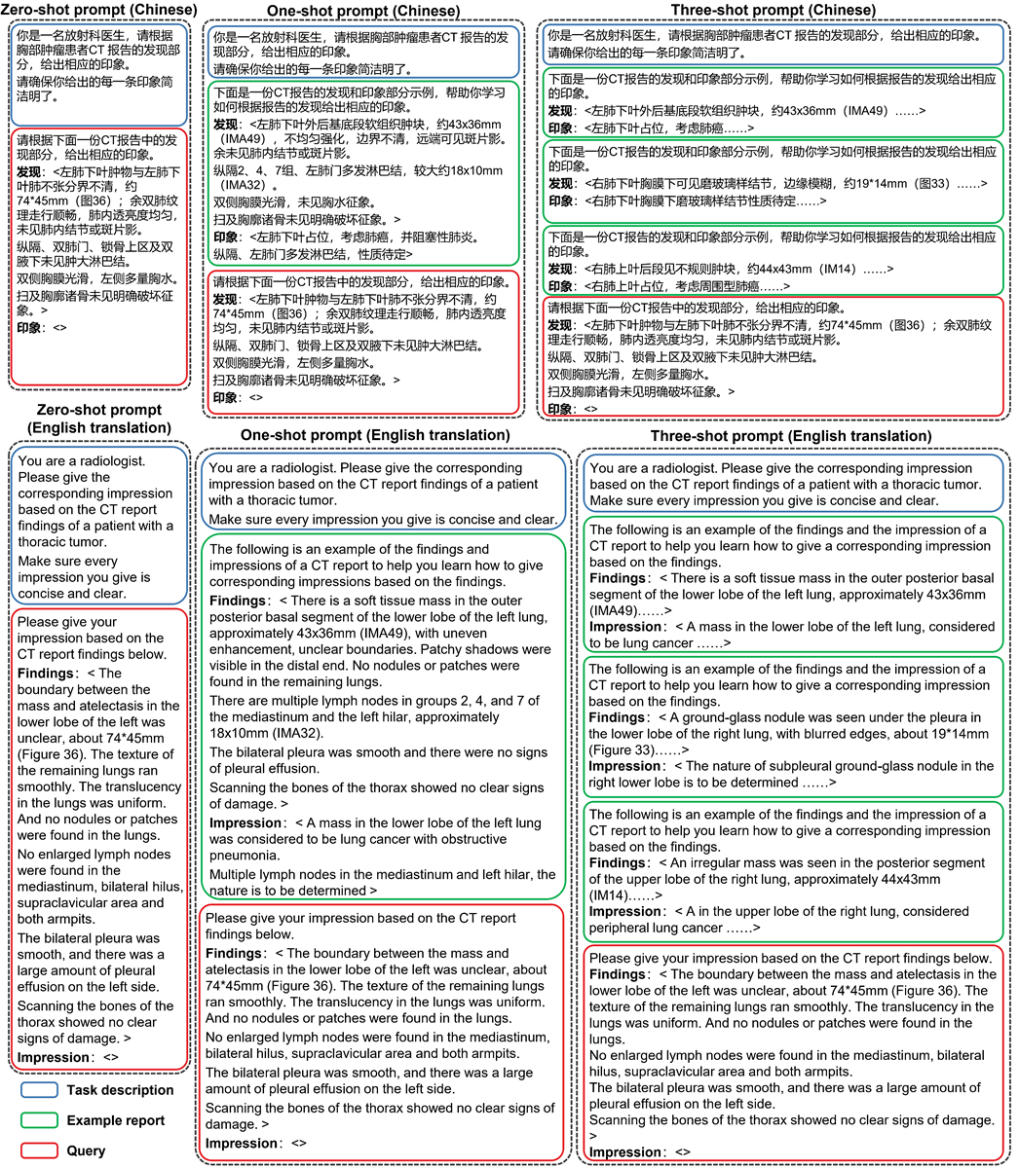
Zero-shot, one-shot, and three-shot prompt examples in Chinese and English. The prompts translated into English are provided for the reader’s benefit, and these translations were not used in the study.

To explore the capability of LLMs to summarize the impression in a zero-shot or few-shot manner, we first designed the zero-, one-, and three-shot prompts, as shown in Figure 2. The language of the prompts is also listed in Multimedia Appendix 1. The zero-shot prompt is composed of two main components, that is, task description and query. In the task description, we first defined the role of the LLMs as a radiologist and then specified the task of generating impressions based on the findings from CT, PET-CT, or US radiology reports. Additionally, we included an instruction emphasizing the need for concise and clear impressions. In the query section, we formatted the findings of the reports using a “Findings:<xxx>” template and provided an “Impressions:<>” template with an empty placeholder “<>,” where the LLMs are expected to generate and insert the generated impressions. For the few-shot prompts, we inserted one or three example reports between the task description and query sections as the one-shot and three-shot prompts, respectively. The example reports were formatted using the same “Findings:<xxx>” and “Impressions:<xxx>” templates to ensure that LLMs can learn and adhere to the desired response format. These prompts allowed us to investigate the impact of the number of examples on the quality of the impression generation. Note that the example reports were randomly selected from the collected reports and different from the report in the query. Since the maximum input text lengths supported by LLMs are different, to fairly evaluate and compare the performance of LLMs, we did not conduct experiments when some prompts exceed the maximum input text length of LLMs (one-shot PET-CT prompt for ERNIE Bot, ChatGLM_Med, three-shot PET-CT prompt for ERINE Bot, Baichuan, HuatuoGPT, and ChatGLM_Med).

Using the developed prompts, we collected the outputs of the five commercially available LLMs from their corresponding websites manually, and we deployed the four open-source LLMs with the default hyperparameters on our server to obtain their outputs. Note that, besides the summarized impression, the LLMs usually generated some other content such as the findings, future examination advice, and the explanation of the response. To accurately evaluate the generated impressions, we conducted a postprocessing procedure to remove the unrelated content from the outputs to keep the impressions only for further quantitative and human evaluations.

### Automatic Quantitative Evaluation Metrics

#### Overview

In this study, we selected three metrics widely used in text generation research to evaluate the generated impression against the reference impression. The values of these metrics range from 0 to 1, where a higher value indicates a better result. A brief introduction of the metrics is listed below.

#### Bilingual Evaluation Understudy

Bilingual Evaluation Understudy (BLEU) [41] score measures the number of position-independent matches of the n-grams of the candidate with the n-grams of the reference, focusing on the precision of the n-grams.

#### Recall-Oriented Understudy for Gisting Evaluation Using Longest Common Subsequence

Recall-Oriented Understudy for Gisting Evaluation Using Longest Common Subsequence (ROUGE-L) [42] measures the longest common subsequence between the candidate and reference to calculate the longest common subsequence–based F-measure.

#### Metric for Evaluation of Translation With Explicit Ordering

Metric for Evaluation of Translation With Explicit Ordering (METEOR) [43] measures the harmonic mean of precision and recall calculated based on the mapping between unigrams with the least number of crosses by exact, stemming, and synonym matching.

### Human Semantic Evaluation Metrics

#### Overview

Although the automatic quantitative evaluation metrics above have shown some correlations with human judgments, they are not sufficient to evaluate the difference between the generated and reference impressions in semantics. Therefore, we defined five human evaluation metrics, that is, (1) correctness, (2) completeness, (3) conciseness, (4) verisimilitude, and (5) replaceability, in this study to evaluate the semantics of the generated impressions. The definitions are listed below. We recruited three clinical experts (SZ, BL, and QL) to annotate the generated impression. We used a 5-point Likert scale for each evaluation metric. A higher value indicates a better result. Note that during human semantic evaluation, the clinical experts could only view the generated impressions, reference impressions, and the corresponding findings. For each evaluated impression, they were blinded to both the identity of the LLM that generated the impression and the type of prompt used (zero-shot, one-shot, or three-shot prompt).

#### Completeness

Completeness measures how completely the information in the generated impression covers the information in the reference impression. The five answer statements for the 5-point Likert scale of completeness are 1=Very incomplete, 2=Relatively incomplete, 3=Neutral, 4=Relatively completeness, and 5=Very correct.

#### Correctness

Correctness measures how correct the information in the generated impression is compared to the information in the reference impression. The five answer statements for the 5-point Likert scale of correctness are 1=Very incorrect, 2=Relatively incorrect, 3=Neutral, 4=Relatively correct, and 5=Very correct.

#### Conciseness

Conciseness measures how much redundant information is in the generated impression. The five answer statements for the 5-point Likert scale of conciseness are 1=Very redundant, 2=Relatively redundant, 3=Neutral, 4=Relatively concise, and 5=Very concise.

#### Verisimilitude

Verisimilitude measures how similar the generated impression is to the reference impression in readability, grammar, and writing style. The five answer statements for the 5-point Likert scale of verisimilitude are 1=Very fake, 2=Relatively fake, 3=Neutral, 4=Relatively verisimilar, and 5=Very verisimilar.

#### Replaceability

Replaceability measures whether the generated impression can replace the reference impression. The five answer statements for the 5-point Likert scale of replaceability are 1=Very irreplaceable, 2=Relatively irreplaceable, 3=Neutral, 4=Relatively replaceable, and 5=Very replaceable.

### Ethical Considerations

Ethical approval for this study was granted by the Ethics Committee of Peking University Cancer Hospital (2022KT128) before this study. Informed consent was waived due to the retrospective design of this study. Data were stored securely with access restricted to the research team, and we removed all identifying information from the collected radiology reports before analysis. No personally identifiable information was included in the study or supplementary materials.

### Statistical Analysis

In this study, we used the Mann-Whitney *U* test to compare the human semantic evaluation results between zero-shot, one-shot, and three-shot prompt strategies. A *P*<.05 was considered statistically significant. The statistical analyses were conducted using the Scipy 1.7.3 Python package.

## Results

### Automatic Quantitative Evaluation Results

**Figure 3:**
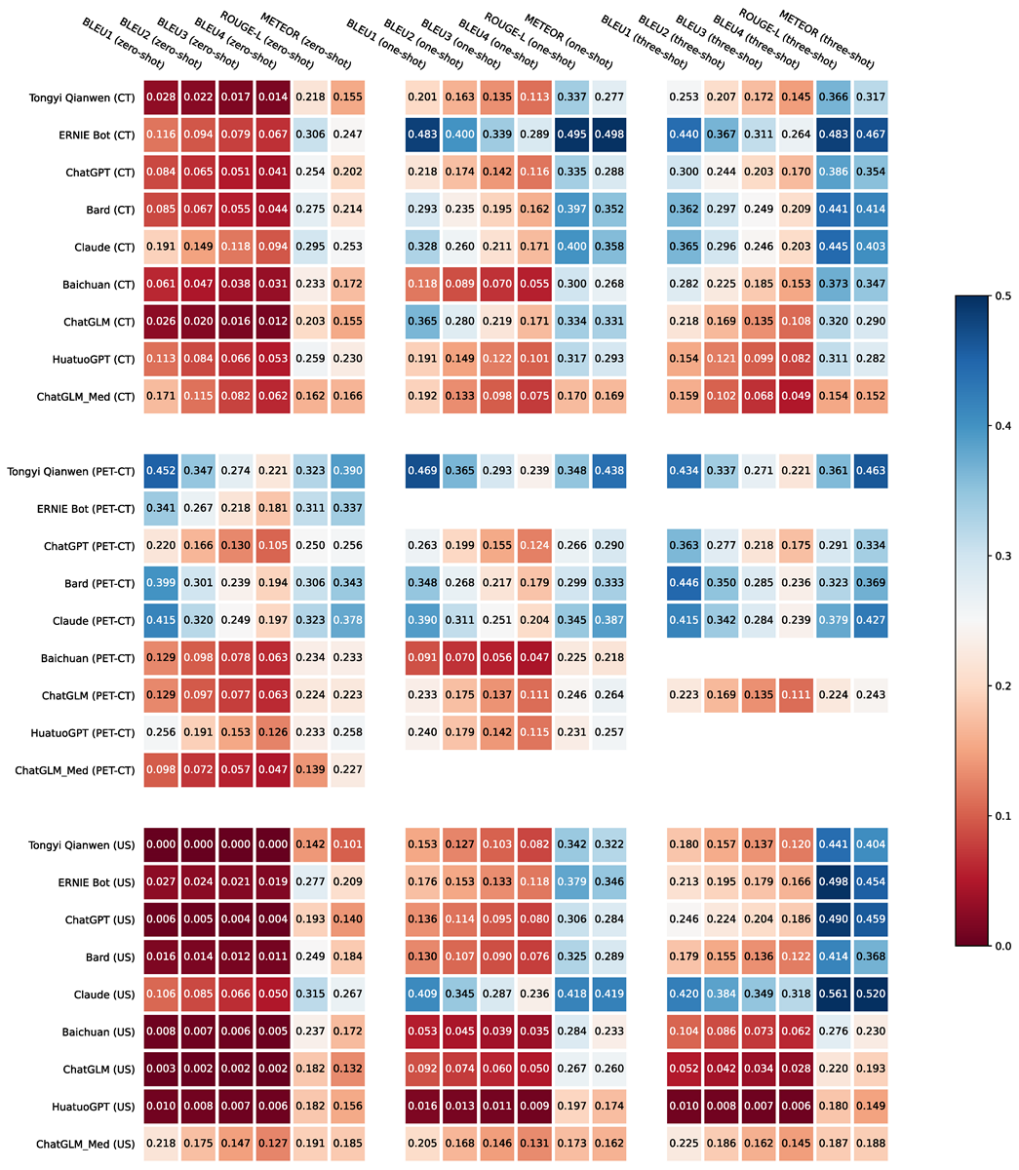
Heatmap visualization of automatic quantitative assessment for the generated CT, PET-CT, and US impressions. BLEU: Bilingual Evaluation Understudy; CT: computed tomography; METEOR: Metric for Evaluation of Translation With Explicit Ordering; PET: positron emission tomography; ROUGE-L: Recall-Oriented Understudy for Gisting Evaluation Using Longest Common Subsequence; US: ultrasound.

Table 2 shows the BLEU, ROUGE-L, and METEOR values of the nine LLMs. We noticed that the ERNIE Bot obtained the overall best results for CT impression summarization, Tongyi Qianwen achieved the best performance for PET-CT impression summarization, and Claude showed the best performance for US impression summarization. Note that the best LLMs are all commercially available models. Moreover, all the best results were obtained based on the one-shot or few-shot prompts, indicating LLMs can learn from the example reports in the prompt to generate better impressions, but more is not necessarily better. Figure 3 illustrates the experimental results more intuitively.

**Table 2:** Automatic quantitative evaluation results of the generated CT^a^, PET^b^-CT, and US^c^ impressions.

Report type, prompt type, and model				BLEU^d^1		BLEU2		BLEU3		BLEU4		ROUGE-L^e^		METEOR^f^
**CT**														
	**Zero-shot**													
		Tongyi Qianwen	0.028		0.022		0.017		0.014		0.218		0.155	
		ERNIE Bot	0.116		0.094		0.079		0.067		0.306		0.247	
		ChatGPT	0.084		0.065		0.051		0.041		0.254		0.202	
		Bard	0.085		0.067		0.055		0.044		0.275		0.214	
		Claude	0.191		0.149		0.118		0.094		0.295		0.253	
		Baichuan	0.061		0.047		0.038		0.031		0.233		0.172	
		ChatGLM	0.026		0.020		0.016		0.012		0.203		0.155	
		HuatuoGPT	0.113		0.084		0.066		0.053		0.259		0.230	
		ChatGLM-Med	0.171		0.115		0.082		0.062		0.162		0.166	
	**One-shot**													
		Tongyi Qianwen	0.201		0.163		0.135		0.113		0.337		0.277	
		ERNIE Bot	0.483		0.400		0.339		0.289		0.495		0.498	
		ChatGPT	0.218		0.174		0.142		0.116		0.335		0.288	
		Bard	0.293		0.235		0.195		0.162		0.397		0.352	
		Claude	0.328		0.260		0.211		0.171		0.400		0.358	
		Baichuan	0.118		0.089		0.070		0.055		0.300		0.268	
		ChatGLM	0.365		0.280		0.219		0.171		0.334		0.331	
		HuatuoGPT	0.191		0.149		0.122		0.101		0.317		0.293	
		ChatGLM-Med	0.192		0.133		0.098		0.075		0.170		0.169	
	**Three-shot**													
		Tongyi Qianwen	0.253		0.207		0.172		0.145		0.366		0.317	
		ERNIE Bot	0.440		0.367		0.311		0.264		0.483		0.467	
		ChatGPT	0.300		0.244		0.203		0.170		0.386		0.354	
		Bard	0.362		0.297		0.249		0.209		0.441		0.414	
		Claude	0.365		0.296		0.246		0.203		0.445		0.403	
		Baichuan	0.282		0.225		0.185		0.153		0.373		0.347	
		ChatGLM	0.218		0.169		0.135		0.108		0.320		0.290	
		HuatuoGPT	0.154		0.121		0.099		0.082		0.311		0.282	
		ChatGLM-Med	0.159		0.102		0.068		0.049		0.154		0.152	
**PET-CT**														
	**Zero-shot**													
		Tongyi Qianwen	0.452		0.347		0.274		0.221		0.323		0.390	
		ERNIE Bot	0.341		0.267		0.218		0.181		0.311		0.337	
		ChatGPT	0.220		0.166		0.130		0.105		0.250		0.256	
		Bard	0.399		0.301		0.239		0.194		0.306		0.343	
		Claude	0.415		0.320		0.249		0.197		0.323		0.378	
		Baichuan	0.129		0.098		0.078		0.063		0.234		0.233	
		ChatGLM	0.129		0.097		0.077		0.063		0.224		0.223	
		HuatuoGPT	0.256		0.191		0.153		0.126		0.233		0.258	
		ChatGLM-Med	0.098		0.072		0.057		0.047		0.139		0.227	
	**One-shot**													
		Tongyi Qianwen	0.469		0.365		0.293		0.239		0.348		0.438	
		ERNIE Bot	—^g^		—		—		—		—		—	
		ChatGPT	0.263		0.199		0.155		0.124		0.266		0.290	
		Bard	0.348		0.268		0.217		0.179		0.299		0.333	
		Claude	0.390		0.311		0.251		0.204		0.345		0.387	
		Baichuan	0.091		0.070		0.056		0.047		0.225		0.218	
		ChatGLM	0.233		0.175		0.137		0.111		0.246		0.264	
		HuatuoGPT	0.240		0.179		0.142		0.115		0.231		0.257	
		ChatGLM-Med	—		—		—		—		—		—	
	**Three-shot**													
		Tongyi Qianwen	0.434		0.337		0.271		0.221		0.361		0.463	
		ERNIE Bot	—		—		—		—		—		—	
		ChatGPT	0.363		0.277		0.218		0.175		0.291		0.334	
		Bard	0.446		0.350		0.285		0.236		0.323		0.369	
		Claude	0.415		0.342		0.284		0.239		0.379		0.427	
		Baichuan	—		—		—		—		—		—	
		ChatGLM	0.223		0.169		0.135		0.111		0.224		0.243	
		HuatuoGPT	—		—		—		—		—		—	
		ChatGLM-Med	—		—		—		—		—		—	
**US**														
	**Zero-shot**													
		Tongyi Qianwen	0.000		0.000		0.000		0.000		0.142		0.101	
		ERNIE Bot	0.027		0.024		0.021		0.019		0.277		0.209	
		ChatGPT	0.006		0.005		0.004		0.004		0.193		0.140	
		Bard	0.016		0.014		0.012		0.011		0.249		0.184	
		Claude	0.106		0.085		0.066		0.050		0.315		0.267	
		Baichuan	0.008		0.007		0.006		0.005		0.237		0.172	
		ChatGLM	0.003		0.002		0.002		0.002		0.182		0.132	
		HuatuoGPT	0.010		0.008		0.007		0.006		0.182		0.156	
		ChatGLM-Med	0.218		0.175		0.147		0.127		0.191		0.185	
	**One-shot**													
		Tongyi Qianwen	0.153		0.127		0.103		0.082		0.342		0.322	
		ERNIE Bot	0.176		0.153		0.133		0.118		0.379		0.346	
		ChatGPT	0.136		0.114		0.095		0.080		0.306		0.284	
		Bard	0.130		0.107		0.090		0.076		0.325		0.289	
		Claude	0.409		0.345		0.287		0.236		0.418		0.419	
		Baichuan	0.053		0.045		0.039		0.035		0.284		0.233	
		ChatGLM	0.092		0.074		0.060		0.050		0.267		0.260	
		HuatuoGPT	0.016		0.013		0.011		0.009		0.197		0.174	
		ChatGLM-Med	0.205		0.168		0.146		0.131		0.173		0.162	
	**Three-shot**													
		Tongyi Qianwen	0.180		0.157		0.137		0.120		0.441		0.404	
		ERNIE Bot	0.213		0.195		0.179		0.166		0.498		0.454	
		ChatGPT	0.246		0.224		0.204		0.186		0.490		0.459	
		Bard	0.179		0.155		0.136		0.122		0.414		0.368	
		Claude	0.420		0.384		0.349		0.318		0.561		0.520	
		Baichuan	0.104		0.086		0.073		0.062		0.276		0.230	
		ChatGLM	0.052		0.042		0.034		0.028		0.220		0.193	
		HuatuoGPT	0.010		0.008		0.007		0.006		0.180		0.149	
		ChatGLM-Med	0.225		0.186		0.162		0.145		0.187		0.188	

^a^CT: computed tomography.

^b^PET: positron emission tomography.

^c^US: ultrasound.

^d^BLEU: Bilingual Evaluation Understudy.

^e^ROUGE-L: Recall-Oriented Understudy for Gisting Evaluation Using Longest Common Subsequence.

^f^METEOR: Metric for Evaluation of Translation With Explicit Ordering.

^g^The length of some prompts exceeds the maximum input text length of the large language model.

### Human Semantic Evaluation Results

#### Quality Evaluation

As the LLMs may produce undesired context, we first manually reviewed the quality of the generated impressions. After review, we summarized five types of errors in generated impressions, that is, refuse-to-answer, truncated-output, repeated-output, no-output, and English-output errors.

All five commercially available LLMs produced high-quality impressions with no truncated-output, repeated-output, or English-output errors. Only the Bard model refused to provide answers for 4 PET-CT impression summarization prompts in one-shot and three-shot manners, respectively.

Different from the commercially available LLMs, the quality of generated impressions varied a lot among the four open-source LLMs. Table 3 shows the errors of the open-source LLMs. The Baichuan model achieved high-quality results, where only one output had the no-output error. The ChatGLM model also achieved good results when using zero-shot prompts, with only one truncated-output error. However, the ChatGLM model obtained many no-output errors when using few-shot prompts. Most of the no-output errors were due to the direct copy of the query section in the prompt but no generated impression. The two medical LLMs, HuatuoGPT and ChatGLM-Med, experienced serious errors in summarizing impressions. HuatuoGPT obtained truncated output and repeated-output errors in over 40% of PET-CT impression summarization tasks. Although the percentage of errors in the CT and US impression summarization decreased, 13.67% and 18.67% of summarized impressions still contained repeated-output errors, respectively. Note that HuatuoGPT was more prone to obtain repeated output errors when using few-shot prompts. The ChatGLM-Med obtained truncated output, repeated-output, and no-output errors in over 40% of generated PET-CT impressions, and 13.33% of generated CT impressions and 22.67% of generated US impressions had truncated-output, repeated-output, and no-output errors.

**Table 3:** Quality assessment results of the open-source LLMs^a^ for CT^b^, PET^c^-CT, and US^d^ impression generation.

Report type, model, and prompt type				Normal (%)		English-output (%)		No-output (%)		Repeated-output (%)		Truncated-output (%)
**CT**												
	**Baichuan**											
		Zero-shot	100		0		0		0		0	
		One-shot	100		0		0		0		0	
		Three-shot	99		0		1		0		0	
	**ChatGLM**											
		Zero-shot	98		2		0		0		0	
		One-shot	89		0		11		0		0	
		Three-shot	94		0		6		0		0	
	**HuatuoGPT**											
		Zero-shot	96		0		0		4		0	
		One-shot	82		0		0		18		0	
		Three-shot	81		0		0		19		0	
	**ChatGLM-Med**											
		Zero-shot	90		0		1		0		9	
		One-shot	87		0		3		0		10	
		Three-shot	83		0		6		1		10	
**PET-CT**												
	**Baichuan**											
		Zero-shot	100		0		0		0		0	
		One-shot	100		0		0		0		0	
		Three-shot	—^e^		—		—		—		—	
	**ChatGLM**											
		Zero-shot	99		0		0		0		1	
		One-shot	99		1		0		0		0	
		Three-shot	90		2		6		0		2	
	**HuatuoGPT**											
		Zero-shot	62		0		0		28		10	
		One-shot	46		0		0		35		19	
		Three-shot	—		—		—		—		—	
	**ChatGLM-Med**											
		Zero-shot	51		0		23		14		12	
		One-shot	—		—		—		—		—	
		Three-shot	—		—		—		—		—	
**US**												
	**Baichuan**											
		Zero-shot	100		0		0		0		0	
		One-shot	100		0		0		0		0	
		Three-shot	100		0		0		0		0	
	**ChatGLM**											
		Zero-shot	100		0		0		0		0	
		One-shot	99		0		1		0		0	
		Three-shot	84		1		15		0		0	
	**HuatuoGPT**											
		Zero-shot	97		0		0		3		0	
		One-shot	71		0		0		28		1	
		Three-shot	74		0		1		25		0	
	**ChatGLM-Med**											
		Zero-shot	77		0		11		3		9	
		One-shot	82		0		6		1		11	
		Three-shot	73		0		10		7		10	

^a^LLM: large language model.

^b^CT: computed tomography.

^c^PET: positron emission tomography.

^d^US: ultrasound.

^e^The length of some prompts exceeds the maximum input text length of the LLM.

#### Semantic Evaluation

**Figure 4:**
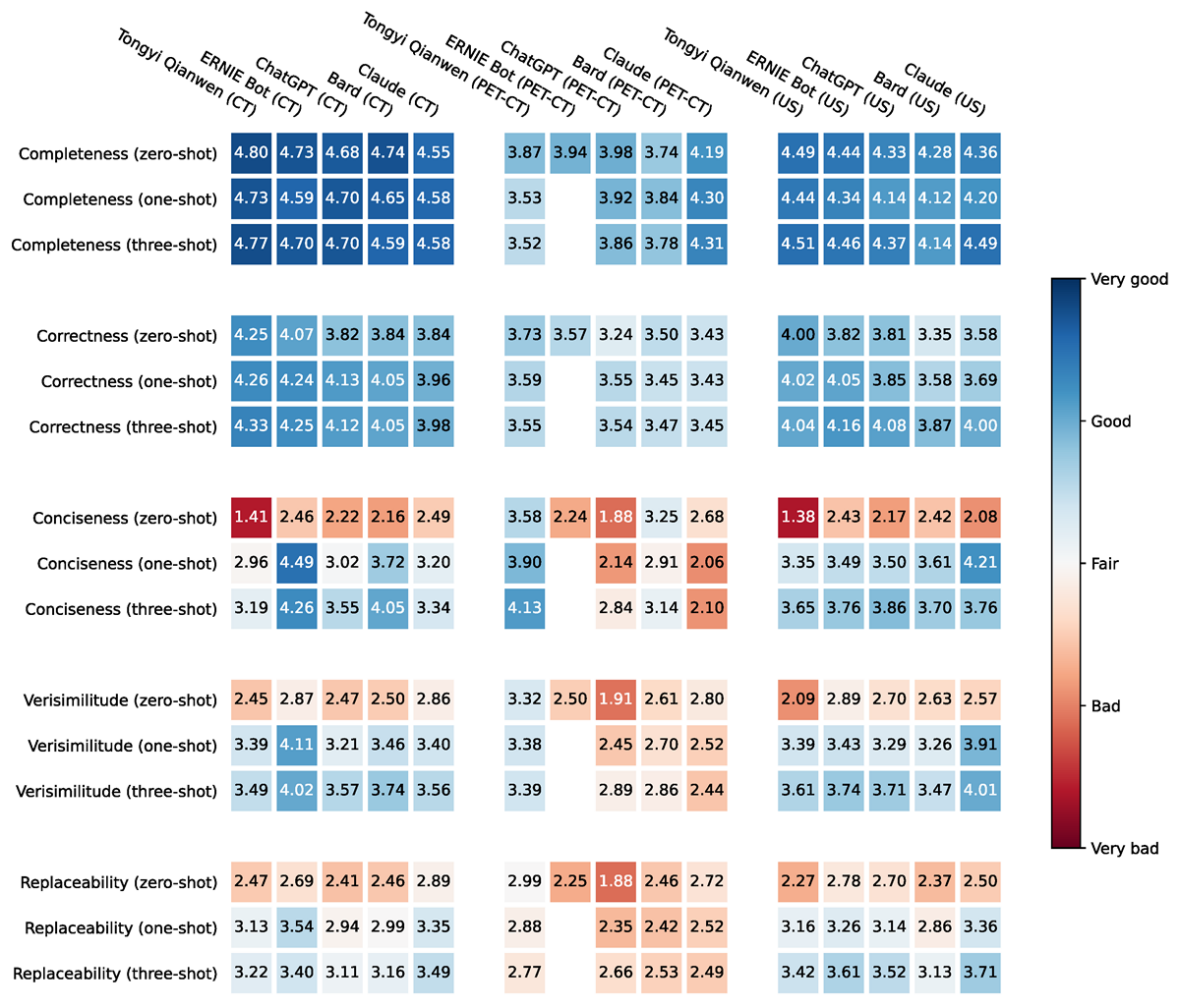
Heatmap visualization of averaged human semantic assessment for the generated CT, PET-CT, and US impressions. CT: computed tomography; PET: positron emission tomography; US: ultrasound.

Based on the automatic quantitative and manual quality evaluation, we noted that the five commercially available LLMs achieved better impression summarization than the four open-source LLMs with higher BLEU, ROUGE-L, and METEOR values and better generation qualities. Therefore, we further evaluated the semantics of the generated impressions of the five commercially available LLMs. We defined five human evaluation metrics: (1) completeness, (2) correctness, (3) conciseness, (4) verisimilitude, and (5) replaceability. The human evaluation results are shown in Table 4. Figure 4 illustrates the human evaluation results in a more intuitive way.

In terms of completeness, the generated CT and US impressions were generally better than the generated PET-CT impressions. Clinical experts rated all of the generated CT and US impressions by different LLMs as between “Relatively complete” and “Very complete” (the best scores were 4.80 for CT impressions and 4.51 for US impressions), while the PET-CT impressions generated by all but Claude were only close to “Relatively complete.” By comparing different prompt types, we noted that some impressions using few-shot prompts achieved lower completeness scores than the impressions using zero-shot prompts, but the decrease was limited.

In terms of correctness, the generated CT and US impressions also achieved good results (the best scores were 4.33 for CT impressions and 4.16 for US impressions), which were between “Relatively correct” and “Very correct.” The generated PET-CT impressions obtained 3.73 for correctness, not reaching the “Relatively correct” level. We also noted that, when using few-shot prompts, the generated CT and US impressions had higher correctness scores, but lower correctness scores for the generated PET-CT impressions compared with using zero-shot prompts.

In terms of conciseness, the generated CT, PET-CT, and US impressions obtained good results. Clinicians rated the generated CT, PET-CT, and US impressions as between “Relatively concise” and “Very concise” (the best scores were 4.49 for CT impressions, 4.13 for PET-CT impressions, and 4.21 for US impressions). When using few-shot prompts, the conciseness scores of the generated impressions achieved significant improvements compared with the generated impressions using zero-shot prompts.

In terms of verisimilitude, the generated CT and US impressions scored more than 4 points (the best scores were 4.11 for CT impressions and 4.01 for US impressions), while the generated PET-CT impressions scored between “Neutral” and “Relatively verisimilar” (the best score was 3.39 for PET-CT impressions). Note that using few-shot prompts can also improve the verisimilitude of the generated impressions significantly.

To comprehensively evaluate the semantics of the generated impressions, clinical experts rated the replaceability of these impressions. We found that the impressions generated by LLMs were not yet at the level that can replace manually written impressions. The generated CT and US impressions only achieved the replaceability scores of 3.54 and 3.71, which were between “Neutral” and “Relatively replaceable,” while the generated PET-CT impressions have an even lower replaceability score of 2.99, which is only close to “Neutral.”

**Table 4:** Averaged human semantic evaluation results of the generated CT^a^, PET^b^-CT, and US^c^ impressions.

Report type, metric, and prompt type				Tongyi Qianwen		ERNIE Bot		ChatGPT		Bard		Claude
**CT**												
	**Completeness**											
		Zero-shot	4.80		4.73		4.68		4.74		4.55	
		One-shot	4.73		4.59		4.70		4.65		4.58	
		Three-shot	4.77		4.70		4.70		4.59		4.58	
	**Correctness**											
		Zero-shot	4.25		4.07		3.82		3.84		3.84	
		One-shot	4.26		4.24		4.13		4.05		3.96	
		Three-shot	4.33		4.25		4.12		4.05		3.98	
	**Conciseness**											
		Zero-shot	1.41		2.46		2.22		2.16		2.49	
		One-shot	2.96		4.49		3.02		3.72		3.20	
		Three-shot	3.19		4.26		3.55		4.05		3.34	
	**Verisimilitude**											
		Zero-shot	2.45		2.87		2.47		2.50		2.86	
		One-shot	3.39		4.11		3.21		3.46		3.40	
		Three-shot	3.49		4.02		3.57		3.74		3.56	
	**Replaceability**											
		Zero-shot	2.47		2.69		2.41		2.46		2.89	
		One-shot	3.13		3.54		2.94		2.99		3.35	
		Three-shot	3.22		3.40		3.11		3.16		3.49	
**PET-CT**												
	**Completeness**											
		Zero-shot	3.87		3.94		3.98		3.74		4.19	
		One-shot	3.53		—^d^		3.92		3.84		4.30	
		Three-shot	3.52		—		3.86		3.78		4.31	
	**Correctness**											
		Zero-shot	3.73		3.57		3.24		3.50		3.43	
		One-shot	3.59		—		3.55		3.45		3.43	
		Three-shot	3.55		—		3.54		3.47		3.45	
	**Conciseness**											
		Zero-shot	3.58		2.24		1.88		3.25		2.68	
		One-shot	3.90		—		2.14		2.91		2.06	
		Three-shot	4.13		—		2.84		3.14		2.10	
	**Verisimilitude**											
		Zero-shot	3.32		2.50		1.91		2.61		2.80	
		One-shot	3.38		—		2.45		2.70		2.52	
		Three-shot	3.39		—		2.89		2.86		2.44	
	**Replaceability**											
		Zero-shot	2.99		2.25		1.88		2.46		2.72	
		One-shot	2.88		—		2.35		2.42		2.52	
		Three-shot	2.77		—		2.66		2.53		2.49	
**US**												
	**Completeness**											
		Zero-shot	4.49		4.44		4.33		4.28		4.36	
		One-shot	4.44		4.34		4.14		4.12		4.20	
		Three-shot	4.51		4.46		4.37		4.14		4.49	
	**Correctness**											
		Zero-shot	4.00		3.82		3.81		3.35		3.58	
		One-shot	4.02		4.05		3.85		3.58		3.69	
		Three-shot	4.04		4.16		4.08		3.87		4.00	
	**Conciseness**											
		Zero-shot	1.38		2.43		2.17		2.42		2.08	
		One-shot	3.35		3.49		3.50		3.61		4.21	
		Three-shot	3.65		3.76		3.86		3.70		3.76	
	**Verisimilitude**											
		Zero-shot	2.09		2.89		2.70		2.63		2.57	
		One-shot	3.39		3.43		3.29		3.26		3.91	
		Three-shot	3.61		3.74		3.71		3.47		4.01	
	**Replaceability**											
		Zero-shot	2.27		2.78		2.70		2.37		2.50	
		One-shot	3.16		3.26		3.14		2.86		3.36	
		Three-shot	3.42		3.61		3.52		3.13		3.71	

^a^CT: computed tomography.

^b^PET: positron emission tomography.

^c^US: ultrasound.

^d^The length of some prompts exceeds the maximum input text length of the large language model.

When comparing the performances of different LLMs, we noted that Tongyi Qianwen achieved the best results on the PET-CT impression generation task, with the best results in four of the five human evaluation metrics, that is, correctness, conciseness, verisimilitude, and replaceability. ERNIE Bot outperformed the other LLMs on the CT impression generation task with the highest scores in conciseness, verisimilitude, and replaceability, and comparable scores in completeness and correctness. For the US impression generation task, Claude achieved better results than other LLMs in conciseness, verisimilitude, and replaceability and comparable results in completeness and conciseness.

To analyze the evaluation variances between the clinical experts, we also list the evaluation results of each clinical expert in Tables S1-S9 and Figures S1-S3 in Multimedia Appendix 1. Based on the results, we noted that there were differences in the scores of different clinical experts. Clinician I’s scores were relatively low. He or she thought that none of the three types of generated impressions could reach the level of replacing manually written impressions (the best score is 2.97 for US impressions). Clinician II’s scores were in the middle. He or she thought that the generated CT and US impressions were close to replacing manually written impressions (the best scores were 3.99 for CT impressions and 3.82 for US impressions), but the generated PET-CT impressions were just neutral in the replaceability (the best score was 3.02 for PET-CT impressions). Clinician III’s scores were relatively higher than the others. He or she thought the generated PET-CT and CT impressions were close to replacing manually written impressions (the best scores were 4.08 for CT impressions and 3.98 for PET-CT impressions), and the generated US impressions could basically replace the manually written impressions (the best score was 4.56 for US impressions).

Although the absolute values of the scores were different between clinical experts, the changing trends of impression scores under different prompt types were similar. Using few-shot prompts can improve most of the conciseness, verisimilitude, and replaceability scores significantly, but may lead to lower completeness and correctness scores. We also illustrate the significant test results in Figures S4-S12 in Multimedia Appendix 1.

## Discussion

### Principal Results

#### Overview

In this study, we aim to explore the capability of the LLMs in summarizing radiology report impressions. Automatic quantitative and human semantic evaluations were conducted to measure the gap between the generated and reference impressions.

#### Commercially Available LLMs Versus Open Source LLMs

To have a comprehensive evaluation of the state-of-the-art LLMs, in this study, we selected five commercially available LLMs, that is, ChatGPT, Bard, ERNIE Bot, Tongyi Qianwen, and Claude, and four open source LLMs, that is, Baichuan, ChatGLM, HuatuoGPT, and ChatGLM-Med. According to the automatic quantitative evaluation, we noted that the commercially available LLMs outperformed the open-source LLMs. Besides, the open-source LLMs exhibited more output errors in the generated impressions, such as the refuse-to-answer, truncated-output, repeated-output, no-output, and English-output errors. These errors were almost absent in the outputs of the commercially available LLMs. When using few-shot prompts, the commercially available LLMs can provide more benefit than the open-source LLMs, thus achieving higher improvements in the automatic quantitative evaluation metrics. The differences between the performance of commercially available and open-source LLMs may be due to the commercially available LLMs usually having more parameters, using more training data to train, using more advanced closed-source algorithms to optimize, and being developed as web applications with better engineering implementations. The gap between the commercially available and open-source LLMs indicates that more computing resources and specialized engineering groups are critical for better LLMs, which has become the main obstacle for most research groups.

#### No Best Model for All Impression Summarization Tasks

Based on the evaluation results, no single LLM can achieve the best results in all impression summarization tasks. Tongyi Qianwen, ERNIE Bot, and Claude achieved the best overall performance in the PET-CT, CT, and US impression summarization tasks, respectively. Although the experimental results indicate that the evaluated LLMs are very competitive with each other and no one can outperform others in all impression summarization tasks significantly, we noted that the LLMs optimized specifically for Chinese like Tongyi Qianwen and ERNIE Bot achieved better results in CT and PET-CT impression summarization than LLMs such as ChatGPT, Bard, and Claude. This finding suggests the necessity to build LLMs for specific languages, which can achieve better performance on language-specific tasks. Besides, we also observed that the performance of the LLMs varied across different report types, particularly in terms of BLEU score. In Figure 3, the BLEU scores for US impressions were significantly lower than those for CT and PET-CT impressions. The primary reason for this discrepancy was that the reference US impressions were much shorter than the reference CT and PET-CT impressions, resulting in fewer character matches between the generated and reference impressions. When using the zero-shot prompt, the generated US impressions were much longer than the reference impressions, leading to extremely low BLEU scores. Although the use of few-shot prompts made the generated US impressions more concise, the BLEU scores remained lower than those for CT and PET-CT due to the fewer matched characters.

#### Effect of the Few-Shot Prompt

In this study, we also explored the effect of the few-shot prompt on impression summarization. Based on the experimental results, we noted that the few-shot prompt can significantly improve the performance of LLMs on all automatic quantitative evaluation metrics and some human evaluation metrics, including conciseness, verisimilitude, and replaceability. For correctness and completeness, using few-shot prompts may lead to some performance degradation, but usually not significant. When further comparing the performance of LLMs using one-shot and three-shot prompts, we found that more examples did not necessarily generate better impressions. For example, Tongyi Qianwen achieved the best BLEU values when using one-shot prompts and the best ROUGE-L and METEOR values when using three-shot prompts for PET-CT impression summarization. ERINE Bot outperformed the other LLMs in automatic metrics for CT impression summarization when using one-shot prompts but Claude achieved the best automatic metrics for US impression summarization when using three-shot prompts. Although there was an overall trend that using few-shot prompts will improve the performance of LLMs in generating impressions, it seems unclear how many examples a prompt should include to be most effective.

#### Clinical Application

Note that to evaluate the semantics of the generated impressions, we first extracted the impressions from the generated text manually and then conducted the human evaluation. Therefore, the current experimental results may be higher than those obtained by evaluating the original outputs. We list the automatic quantitative results in Tables S10-S12 and Figure S13 in Multimedia Appendix 1. We also show the difference in results between using the extracted impressions and original outputs in Figure S14 in Multimedia Appendix 1. We can note that all results obtained by evaluating extracted impressions were higher than those on original outputs. However, among all LLMs, Tongyi Qianwen, ERNIE Bot, and ChatGPT showed small differences between these results, indicating they can follow the instructions well to generate the text we desire. Although Bard and Claude achieved comparable performance based on the extracted impressions, its original outputs contained much more impression-unrelated content, reducing its usability in summarizing impressions in real clinical practice.

According to the evaluation of clinical experts, the impressions generated by the LLMs cannot directly substitute for those written by radiologists. However, using LLMs to summarize clinical text like radiology findings is still valuable. First, it can help clinicians improve the efficiency of writing clinical documents. In clinical practice, writing clinical documents like radiology reports, admission records, progress notes, and discharge summaries has become a heavy burden for radiologists and clinicians. To alleviate this problem, we can use the LLMs to summarize the related structured or unstructured electronic health records as a preliminary clinical note, and then the clinicians conduct the final review. Moreover, LLMs also have the potential to combine the abnormal findings from multiple types of reports to provide comprehensive evaluations like cancer staging, which can help clinicians assess the patient’s status better. For patients with cancer who usually undergo a long diagnosis and treatment process, we can also use the LLMs to summarize the whole diagnosis and treatment timeline, which is very important and valuable for the development of the next treatment plan. Second, the impressions generated by LLMs demonstrated a high level of completeness and correctness, with evaluation scores ranging from 4.0=good to 5.0=very good. As a result, clinicians may find it useful to reference the diagnoses or suspicions of abnormal findings suggested by LLMs before making their own judgments. However, whether LLMs can really help clinicians with different experiences to make more accurate diagnoses remains unclear, requiring further investigation in future studies. Third, we can use LLMs to facilitate the research. Based on the summarization ability, LLMs can effectively extract key information from clinical documents to identify eligible patients for specific studies.

### Comparisons to Prior Work

In this study, we investigated the performance of widely used commercial and open-source LLMs in summarizing Chinese radiology report impressions for lung cancer. Based on our analysis, we found a gap between the impressions generated by LLMs and those written by radiologists. Previous research has also explored the application of LLMs in clinical text summarization. For instance, Liu et al [22] assessed 29 different LLMs for generating impressions from radiological reports, using open-source datasets such as the MIMIC-CXR and OpenI datasets. However, their study was limited to English x-ray reports and relied solely on automatic quantitative evaluation. Sun et al [23] evaluated GPT-4 for radiological impression generation, introducing four human semantic metrics, that is, coherence, comprehensiveness, factual consistency, and harmfulness, to assess the generated impressions. They found that radiologists outperformed GPT-4 in radiology report generation. Despite incorporating human semantic evaluation, their analysis was only confined to 50 English chest radiograph reports. Van Veen et al [2] conducted a comprehensive evaluation of clinical text summarization, encompassing various radiology reports, progress notes, doctor-patient conversations, and patient questions. They also fine-tuned LLMs for better performance and they concluded that LLMs can outperform medical experts in multiple clinical text summarization tasks. However, their evaluation was restricted to zero-shot prompts and exclusively involved English clinical texts.

Compared with prior works, this study is the first to evaluate LLMs in summarizing three types of Chinese radiology report impressions, that is, CT, PET-CT, and US, and to explore the impact of different prompting strategies, that is, zero-shot, one-shot, and few-shot, on generation performance. Significantly, we conducted a large-scale human semantic evaluation of the generated impressions, providing more robust and convincing results. This study better demonstrates the capabilities of LLMs in non-English clinical text summarization, offering valuable insights into their applicability in diverse linguistic and clinical contexts.

### Limitations

To comprehensively evaluate the LLMs’ impression summarizing ability, we selected three types of radiology reports, that is, PET-CT, CT, and US reports. We should note that all reports were from patients with lung cancer treated in a single medical center, which indicates that the patient population was homogeneous and the writing style of the reports was relatively uniform. So, the results in this study may differ from the average performance of LLMs in summarizing the impressions of reports from patients with different diseases or medical centers. In the future, we will try to collect more radiology reports from different patients and medical centers to evaluate the LLMs to obtain more robust results.

The most important contribution of this study is that we invited three clinical experts to manually evaluate the impressions generated by the LLMs from the point of view of semantics so that we could find out the gap between the reports generated by LLMs and those written by radiologists. However, manual evaluation is time-consuming and tedious, which is the biggest obstacle for large-scale evaluation. Therefore, in this study, we only evaluated 100 generated impressions for each report type. Moreover, the number of clinicians involved in this study was also limited. As we know, the subjectivities of clinicians may vary based on their expertise and background. For example, an oncologist, a radiologist, and a pulmonologist may have different preferences for the radiology impressions of a patient with lung cancer. Furthermore, for complex diseases like lung cancer that typically require multidisciplinary treatment, surgical oncologists, radiation oncologists, and medical oncologists may prioritize different information. In this study, we only recruited three clinical experts, that is, two thoracic surgeons and one radiologist, and just reported their individual evaluation results in the supplement but did not delve into the differences in their preferences and subjectivities. In the future, we will try to recruit more clinicians with different expertise from different departments and conduct subgroup analyses to explore their different views on the impressions generated by LLMs.

Currently, LLMs are updated very quickly. Since human evaluation is very time-consuming, we cannot perform real-time human evaluation of the latest LLMs. In the future, we will try to evaluate the latest LLMs and compare them with their previous versions to find out the changes in the performance of impression summarization of radiology reports. Moreover, many LLMs have demonstrated competitive performance on various medical tasks, such as medical question answering, information extraction, and medical literature summarization. However, to the best of our knowledge, this study is the first to investigate the capability of LLMs in summarizing Chinese radiology impressions. Before this study, only a few studies explored this topic, and those were focused on English reports. Although the languages and types of reports are different, some of our findings align with previous studies, such as comparable ROUGE-L scores. However, there are also some differences, such as different expert opinions on the generated impressions. Due to the constraints of human evaluation, we can only choose a limited number of LLMs to evaluate in this study, excluding several notable LLMs like Med-PaLM and LLaVA-Med. In future work, we plan to expand our evaluation to include more models to draw a more comprehensive picture of LLMs for radiology impression summarization. Furthermore, fine-tuning LLMs with localized data or designing more specialized prompts, such as using the chain-of-thought strategy or requiring output in JSON format, may improve the quality of generated impressions and reduce irrelevant content. We will try to explore the effectiveness of these techniques in the future.

### Conclusions

In this study, we explore the capability of LLMs in summarizing radiology report impressions using automatic quantitative and human evaluations. The experimental results indicate that there is a gap between the impressions generated by LLMs and those written by radiologists. LLMs achieved great performance in completeness and correctness but were not good in conciseness and verisimilitude. Although the few-shot prompt could improve the LLMs’ performance in conciseness and verisimilitude, clinicians still believed that the LLMs could not replace the radiologist in summarizing impressions, especially for PET-CT reports with long findings.

## Appendix

Multimedia Appendix 1 Supplementary material.

## Abbreviations

BLEU: Bilingual Evaluation Understudy
CT: computed tomography
LLM: large language model
METEOR: Metric for Evaluation of Translation With Explicit Ordering
PET: positron emission tomography
RFHL: reinforcement learning from human feedback
ROUGE-L: Recall-Oriented Understudy for Gisting Evaluation Using Longest Common Subsequence
US: ultrasound
