# Supplementary material for: Large Language Models in Summarizing Radiology Report Impressions for Lung Cancer in Chinese: Evaluation Study
Source: J Med Internet Res. 2025 Apr 3;27:e65547. doi: 10.2196/65547 (PMC12006768; doi:10.2196/65547)
Supplement: Multimedia Appendix 1 [file jmir_v27i1e65547_app1.docx]

**A zero-shot prompt example.**

你是一名放射科医生，请根据胸部肿瘤患者CT 报告的发现部分，给出相应的印象。

请确保你给出的每一条印象简洁明了。

请根据下面一份CT报告中的发现部分，给出相应的印象。

发现：<左肺下叶肿物与左肺下叶肺不张分界不清，约74*45mm（图36）；余双肺纹理走行顺畅，肺内透亮度均匀，未见肺内结节或斑片影。

纵隔、双肺门、锁骨上区及双腋下未见肿大淋巴结。

双侧胸膜光滑，左侧多量胸水。

扫及胸廓诸骨未见明确破坏征象。>

印象：<>

English translation version (not used in this study):

You are a radiologist. Please give the corresponding impression based on the CT report findings of a patient with a thoracic tumor.

Make sure every impression you give is concise and clear.

Please give your impression based on the CT report findings below.

Findings：< A mass in the lower lobe of the left lung, indistinctly demarcated from left lower lobe atelectasis, measuring about 74*45mm (Figure 36). The remaining lung markings appear smooth, with uniform lung transparency. No nodules or patchy shadows are observed in the lungs.

No enlarged lymph nodes are seen in the mediastinum, bilateral hilar, supraclavicular regions, or both armpits.

The pleura on both sides is smooth, with a large amount of pleural effusion on the left side.

No clear evidence of destruction in the thoracic bones. >

Impression：<>

**A one-shot prompt example.**

你是一名放射科医生，请根据胸部肿瘤患者CT 报告的发现部分，给出相应的印象。

请确保你给出的每一条印象简洁明了。

下面是一份CT报告的发现和印象部分示例，帮助你学习如何根据报告的发现给出相应的印象。

发现：<左肺下叶外后基底段软组织肿块，约43x36mm（IMA49），不均匀强化，边界不清，远端可见斑片影。余未见肺内结节或斑片影。

纵隔2、4、7组、左肺门多发淋巴结，较大约18x10mm（IMA32）。

双侧胸膜光滑，未见胸水征象。

扫及胸廓诸骨未见明确破坏征象。>

印象：<左肺下叶占位，考虑肺癌，并阻塞性肺炎。

纵隔、左肺门多发淋巴结，性质待定>

请根据下面一份CT报告中的发现部分，给出相应的印象。

发现：<左肺下叶肿物与左肺下叶肺不张分界不清，约74*45mm（图36）；余双肺纹理走行顺畅，肺内透亮度均匀，未见肺内结节或斑片影。

纵隔、双肺门、锁骨上区及双腋下未见肿大淋巴结。

双侧胸膜光滑，左侧多量胸水。

扫及胸廓诸骨未见明确破坏征象。>

印象：<>

English translation version (not used in this study):

You are a radiologist. Please give the corresponding impression based on the CT report findings of a patient with a thoracic tumor.

Make sure every impression you give is concise and clear.

The following is an example of the findings and impressions of a CT report to help you learn how to give corresponding impressions based on the findings.

Findings：< There is a soft tissue mass in the outer posterior basal segment of the lower lobe of the left lung, approximately 43x36mm (IMA49), with uneven enhancement and unclear boundaries. Patchy shadows are visible in the distal end. No nodules or patchy shadows are seen in the remaining lungs.

There are multiple lymph nodes in mediastinal groups 2, 4, and 7 and the left hilar, with the largest being approximately 18x10mm (IMA32).

The pleura on both sides is smooth, and there is no sign of pleural effusion.

No clear evidence of destruction in the thoracic bones. >

Impression：< A mass in the lower lobe of the left lung, likely lung cancer, with obstructive pneumonia.

Multiple lymph nodes in the mediastinum and left hilar, nature to be determined. >

Please give your impression based on the CT report findings below.

Findings：< A mass in the lower lobe of the left lung, indistinctly demarcated from left lower lobe atelectasis, measuring about 74*45mm (Figure 36). The remaining lung markings appear smooth, with uniform lung transparency. No nodules or patchy shadows are observed in the lungs.

No enlarged lymph nodes are seen in the mediastinum, bilateral hilar, supraclavicular regions, or both armpits.

The pleura on both sides is smooth, with a large amount of pleural effusion on the left side.

No clear evidence of destruction in the thoracic bones. >

Impression：<>

**A three-shot prompt example**

你是一名放射科医生，请根据胸部肿瘤患者CT 报告的发现部分，给出相应的印象。

请确保你给出的每一条印象简洁明了。

下面是一份CT报告的发现和印象部分示例，帮助你学习如何根据报告的发现给出相应的印象。

发现：<左肺下叶外后基底段软组织肿块，约43x36mm（IMA49）……>

印象：<左肺下叶占位，考虑肺癌……>

下面是一份CT报告的发现和印象部分示例，帮助你学习如何根据报告的发现给出相应的印象。

发现：<右肺下叶胸膜下可见磨玻璃样结节，边缘模糊，约19*14mm（图33）……>

印象：<右肺下叶胸膜下磨玻璃样结节性质待定……>

下面是一份CT报告的发现和印象部分示例，帮助你学习如何根据报告的发现给出相应的印象。

发现：<右肺上叶后段见不规则肿块，约44x43mm（IM14）……>

印象：<右肺上叶占位，考虑周围型肺癌……>

请根据下面一份CT报告中的发现部分，给出相应的印象。

发现：<左肺下叶肿物与左肺下叶肺不张分界不清，约74*45mm（图36）；余双肺纹理走行顺畅，肺内透亮度均匀，未见肺内结节或斑片影。

纵隔、双肺门、锁骨上区及双腋下未见肿大淋巴结。

双侧胸膜光滑，左侧多量胸水。

扫及胸廓诸骨未见明确破坏征象。>

印象：<>

English translation version (not used in this study):

You are a radiologist. Please give the corresponding impression based on the CT report findings of a patient with a thoracic tumor.

Make sure every impression you give is concise and clear.

The following is an example of the findings and the impression of a CT report to help you learn how to give a corresponding impression based on the findings.

Findings：< There is a soft tissue mass in the outer posterior basal segment of the lower lobe of the left lung, approximately 43x36mm (IMA49)……>

Impression：< A mass in the lower lobe of the left lung, likely lung cancer……>

The following is an example of the findings and the impression of a CT report to help you learn how to give a corresponding impression based on the findings.

Findings：< A ground-glass nodule is seen under the pleura in the lower lobe of the right lung, with unclear margins, about 19*14mm (Figure 33)……>

Impression：< The nature of subpleural ground-glass nodule in the right lower lobe is to be determined ……>

The following is an example of the findings and the impression of a CT report to help you learn how to give a corresponding impression based on the findings.

Findings：< An irregular mass is seen in the posterior segment of the upper lobe of the right lung, approximately 44x43mm (IM14)……>

Impression：< A mass in the upper lobe of the right lung, suspected to be peripheral lung cancer ……>

Please give your impression based on the CT report findings below.

Findings：< A mass in the lower lobe of the left lung, indistinctly demarcated from left lower lobe atelectasis, measuring about 74*45mm (Figure 36). The remaining lung markings appear smooth, with uniform lung transparency. No nodules or patchy shadows are observed in the lungs.

No enlarged lymph nodes are seen in the mediastinum, bilateral hilar, supraclavicular regions, or both armpits.

The pleura on both sides is smooth, with a large amount of pleural effusion on the left side.

No clear evidence of destruction in the thoracic bones. >

Impression：<>

**A real CT report and its corresponding generated impressions by ERNIE-bot.**

*Findings of the example CT report:*

右肺下叶可见不规则肿物，边缘可见少许毛刺，约48*37mm（图39）；余肺野未见肺内结节或斑片影。

纵隔11R淋巴结略增大，约12*8mm（图33）；余纵隔、双肺门、锁骨上区及双腋下未见肿大淋巴结。

双侧胸膜光滑，未见胸水征象。

扫及胸廓诸骨未见明确破坏征象。

English translation version (not used in this study):

An irregular mass is seen in the right lower lung lobe, with slight spiculations at the edges, measuring approximately 48 x 37 mm (Figure 39); no pulmonary nodules or infiltrates are observed in the remaining lung fields.

The 11R mediastinal lymph node is mildly enlarged, measuring approximately 12 x 8 mm (Figure 33); no enlarged lymph nodes are observed in the remaining mediastinum, both lung hila, supraclavicular area, or both axillary regions.

Both pleurae are smooth, with no signs of pleural effusion.

No obvious bone destruction is noted in the scanned ribcage.

*Ground truth impressions of the example CT report:*

右肺下叶不规则肿物，考虑周围型肺癌可能大；

右肺门小淋巴结，转移？建议追查。

English translation version (not used in this study):

An irregular mass in the lower lobe of the right lung is highly suggestive of peripheral lung cancer.

A small lymph node in the right hilar region raises suspicion of metastasis. Further investigation is recommended.

*Impressions generated by ERINE-bot using zero-shot prompt:*

右肺下叶不规则肿物，边缘有少许毛刺，约48*37mm。

纵隔11R淋巴结略增大，约12*8mm。

余肺野、纵隔、双肺门、锁骨上区及双腋下未见肿大淋巴结。

双侧胸膜光滑，未见胸水征象。

胸廓诸骨未见明确破坏征象。

English translation version (not used in this study):

An irregular mass is observed in the lower lobe of the right lung, with a few spiculations at the margins, measuring approximately 4837 mm.

The 11R lymph node in the mediastinum is slightly enlarged, measuring approximately 128 mm.

No enlarged lymph nodes are seen in the remaining lung fields, mediastinum, bilateral hilar regions, supraclavicular areas, or bilateral axillae.

The bilateral pleura are smooth, with no signs of pleural effusion.

No definitive signs of bone destruction are observed in the thoracic skeleton.

*Impressions generated by ERINE-bot using one-shot prompt:*

右肺下叶占位，考虑肺癌可能。纵隔11R淋巴结略增大，性质待定。

English translation version (not used in this study):

A mass in the lower lobe of the right lung is suspected to be lung cancer.

The 11R lymph node in the mediastinum is slightly enlarged, and its nature remains to be determined.

*Impressions generated by ERINE-bot using three-shot prompt:*

右肺下叶不规则肿物，考虑周围型肺癌可能性大。纵隔11R淋巴结略增大，性质待定，建议进一步检查。

English translation version (not used in this study):

An irregular mass in the lower lobe of the right lung is highly suggestive of peripheral lung cancer.

The 11R lymph node in the mediastinum is slightly enlarged, and its nature remains uncertain. Further investigation is recommended.

**A real PET-CT report and its corresponding generated impressions by Tongyi Qianwen.**

*Findings of the example PET-CT report:*

空腹6小时以上，静脉注射显像剂，平静休息60min后行全身PET及CT断层显像，PET图像经衰减校正及重建后，将PET及CT图像行多平面、多幅显示：

左肺上叶软组织结节，伴分叶，约2.8cmx2.5cm大小，放射性分布异常浓聚，最大SUV：8.5，远端伴少许肺不张。余双肺多发微小结节，较大者直径约0.2-0.3cm，均未见明显高摄取。右肺下叶模糊团片影，未见高摄取，左肺下叶基底段肺大泡。

双胸膜光，未见胸腔积液。

纵隔5、6组及左肺门多个淋巴结肿大并放射性分布异常浓聚，最大SUV：7.0，较大者约1.8cmx1.0cm大小。

左4前肋、T8椎体及右耻骨可见异常浓聚灶，最大SUV：7.0，同机CT平扫示局部骨质密度增高。左股骨头骨岛。

大脑形态如常，皮层各叶放射性分布均匀。皮层下各神经核团显影清晰，放射性分布对称。同机CT显示大脑灰质沟回未见明显增宽加深，白质未见明显低密度影，中线无移位。脑室无扩大，基底节区显示对称。小脑显影如常，两侧小脑对称。

口咽部两侧腺体显影对称，鼻咽部无异常放射性浓聚。甲状腺两叶不大，形态可，腺实质内未见异常放射性增高灶。两侧乳腺轻度显影，两侧大致对称，乳腺区未见异常浓聚灶。

胃充盈尚可，胃壁显影如常。肝脏形态可，轮廓光整，肝叶比例正常，肝实质内放射性分布稍欠均匀。肝内外胆管无扩张。胆囊大小正常，密度均匀，胆囊壁无增厚。肝门结构正常。胰腺形态放射性分布尚好，胰管不扩张。脾脏轻度显影，放射性分布均匀。两侧肾脏显影可，右肾见囊性密度结节，直径约0.7cm，放射性分布稀疏缺损，肾盂、肾盏及输尿管无扩张。两侧肾上腺显影大致正常。腹部可见条索状肠影。腹水征阴性。膀胱放射性浓聚如常，膀胱壁无增厚。子宫萎缩，局部无异常放射性浓聚。两侧附件区未见异常放射性摄取。

English translation version (not used in this study):

Fasting for more than 6 hours, followed by intravenous injection of the radiopharmaceutical, with a quiet rest period of 60 minutes before undergoing whole-body PET and CT imaging. PET images were corrected for attenuation and reconstructed, and both PET and CT images were displayed in multiple planes and slices:

A soft tissue nodule in the left upper lung lobe, with lobulation, approximately 2.8 cm x 2.5 cm in size, showing abnormal radioactive uptake, with the maximum SUV of 8.5. A small amount of atelectasis is seen at the distal end. Multiple small nodules are present in both lungs, with the largest measuring about 0.2-0.3 cm in diameter, and no significant high uptake is observed. A blurred mass-like shadow is seen in the right lower lung, with no high uptake. A pulmonary bulla is seen in the basal segment of the left lower lung.

Both pleurae appear clear, with no pleural effusion observed.

Enlarged lymph nodes are present in the 5th and 6th mediastinal groups, as well as at the left hilum of the lung, showing abnormal radioactive uptake, with the maximum SUV of 7.0. The largest node measures about 1.8 cm x 1.0 cm.

Abnormal uptake is seen in the left 4th anterior rib, T8 vertebral body, and right pubic bone, with the maximum SUV of 7.0. A CT scan shows localized increased bone density. A bone island is noted in the left femoral head.

The brain appears normal in morphology, with even radioactive distribution across all cortical lobes. The subcortical neural nuclei are clearly visualized, with symmetrical radioactive distribution. CT imaging shows no significant widening or deepening of the cortical sulci, and no apparent low-density areas in the white matter. The midline is not displaced. No enlargement of the ventricles, and the basal ganglia region shows symmetrical appearance. The cerebellum is normal, with symmetrical visualization on both sides.

Symmetrical uptake is observed in the tonsils on both sides of the oropharynx. No abnormal radioactive concentration is noted in the nasopharynx. The thyroid gland is of normal size and shape, with no abnormal radioactive hot spots within the glandular parenchyma. Mild uptake is noted in both breasts, with symmetry on both sides, and no abnormal concentrations are seen in the breast tissue.

The stomach is adequately filled, with normal wall uptake. The liver has a normal shape and smooth contour, with a normal lobular ratio. Radioactive distribution in the liver parenchyma is slightly uneven. No dilation of the intrahepatic or extrahepatic bile ducts. The gallbladder has a normal size, uniform density, and no wall thickening. The hepatic hilum structure is normal. The pancreas shows normal shape and radioactive distribution, with no duct dilation. Mild uptake is seen in the spleen, with even radioactive distribution. Both kidneys show normal uptake, with a cystic nodule in the right kidney measuring approximately 0.7 cm in diameter, with sparse radioactive distribution and defects. No dilation of the renal pelvis, calyces, or ureters. Both adrenal glands show normal uptake.

The abdominal area shows linear intestinal shadows. No signs of ascites. The bladder shows normal radioactive concentration, with no thickening of the bladder wall. The uterus is atrophic, with no abnormal radioactive uptake in the local area. No abnormal radioactive uptake is noted in the bilateral adnexal regions.

*Ground truth impressions of the example PET-CT report:*

1、①左肺上叶高代谢占位，考虑为肺癌，远端伴少许阻塞性肺不张。

②纵隔5、6组及左肺门多发淋巴结转移。

③双肺多发转移。

④左4前肋、T8椎体及右耻骨骨转移。

2、全脑代谢未见异常，建议必要时行增强MRI扫描以除外微小转移可能。

3、右肺下叶团片影，未见高代谢，建议定期复查。

4、左肺下叶肺大泡；右肾囊肿。左股骨头骨岛。

English translation version (not used in this study):

1.① A hypermetabolic mass in the upper lobe of the left lung is considered to be lung cancer, accompanied by a small amount of obstructive atelectasis distally.

② Multiple lymph node metastases in groups 5 and 6 of the mediastinum and the left pulmonary hilum.

③ Multiple metastases in both lungs.

④ Bone metastases in the left 4th anterior rib, T8 vertebral body, and right pubic bone.

2.No abnormal metabolism was observed in the entire brain. It is recommended to perform an enhanced MRI scan if necessary to rule out the possibility of micro-metastases.

3.A mass-like shadow in the lower lobe of the right lung showed no hypermetabolism, and regular follow-up is recommended.

4.A bulla in the lower lobe of the left lung; a cyst in the right kidney. A bone island in the left femoral head.

*Impressions generated by Tongyi Qianwen using zero-shot prompt:*

1.左肺上叶恶性肿瘤可能，伴左肺下叶基底段肺大泡。

2.双胸膜未见异常，纵隔淋巴结转移可能。

3.多发骨转移：左4前肋、T8椎体及右耻骨病变，考虑骨转移。

4.脑部未见明显异常。

5.鼻咽部、甲状腺、乳腺无明显异常。

6.胃、肝、胆囊、胰腺、脾脏大致正常；右肾囊肿。

7.无腹水，膀胱壁无增厚。子宫萎缩。

English translation version (not used in this study):

1.Suspicion of malignant tumor in the upper lobe of the left lung, accompanied by a pulmonary bulla in the basal segment of the lower lobe of the left lung.

2.No abnormalities observed in the bilateral pleura; possible mediastinal lymph node metastasis.

3.Multiple bone metastases: Lesions in the left 4th anterior rib, T8 vertebral body, and right pubic bone, considered to be bone metastases.

4.No significant abnormalities in the brain.

5.No obvious abnormalities in the nasopharynx, thyroid, or breasts.

6.Stomach, liver, gallbladder, pancreas, and spleen are generally normal; a cyst is present in the right kidney.

7.No ascites, no thickening of the bladder wall. Uterine atrophy is noted.

*Impressions generated by Tongyi Qianwen using one-shot prompt:*

1.左肺上叶软组织结节，考虑为肺癌可能大；

2.双胸膜光，未见胸腔积液；

3.纵隔5、6组及左肺门多个淋巴结肿大并放射性分布异常浓聚，考虑为转移可能性大；

4.左4前肋、T8椎体及右耻骨可见异常浓聚灶，同机CT平扫示局部骨质密度增高，考虑为骨转移可能性大；

5.左股骨头骨岛，良性病变可能。

English translation version (not used in this study):

1.A soft tissue nodule in the upper lobe of the left lung is highly suspected to be lung cancer.

2.The bilateral pleura are clear, with no pleural effusion observed.

3.Multiple enlarged lymph nodes with abnormally intense radioactive uptake are noted in groups 5 and 6 of the mediastinum and the left pulmonary hilum, highly suggestive of metastasis.

4.Abnormal intense uptake foci are seen in the left 4th anterior rib, T8 vertebral body, and right pubic bone. Concurrent non-contrast CT scans show increased local bone density, highly suggestive of bone metastasis.

5.A bone island in the left femoral head is likely a benign lesion.

*Impressions generated by Tongyi Qianwen using three-shot prompt:*

1.左肺上叶软组织结节，伴高代谢，符合肺Ca表现；双肺多发微小结节，未见明显高摄取，建议随访。

2.纵隔5、6组及左肺门多个淋巴结肿大并高代谢，考虑转移；

3.T8椎体及右耻骨异常浓聚灶，考虑转移；左4前肋异常浓聚灶，转移待除外。

4.双侧乳腺轻度显影，两侧大致对称，乳腺区未见异常浓聚灶，建议结合临床。

5.脑部未见明显异常代谢征象；口咽部炎性摄取；左股骨头骨岛；右肾囊性密度结节

English translation version (not used in this study):

1.A soft tissue nodule in the upper lobe of the left lung with high metabolic activity is consistent with the presentation of lung cancer (Ca). Multiple micronodules in both lungs show no significant uptake, and follow-up is recommended.

2.Multiple enlarged lymph nodes with high metabolic activity in groups 5 and 6 of the mediastinum and the left pulmonary hilum are suggestive of metastasis.

3.Abnormal intense uptake foci in the T8 vertebral body and right pubic bone are considered metastatic. An abnormal intense uptake focus in the left 4th anterior rib requires further exclusion of metastasis.

4.Mild bilateral breast imaging is observed, with roughly symmetrical appearance and no abnormal uptake foci in the breast region. Clinical correlation is recommended.

5.No significant abnormal metabolic signs are observed in the brain. Inflammatory uptake is noted in the oropharynx. A bone island is present in the left femoral head, and a cystic density nodule is seen in the right kidney.

**A real US report and its corresponding generated impressions by Claude.**

*Findings of the example US report:*

肝形态、大小正常，回声欠均匀，未见明显占位。

胆系不扩张。

胰腺、双肾及双肾上腺区未见明显占位。

脾不大。

腹主动脉周围未见明显肿大淋巴结。

右锁骨上可见多发低回声小淋巴结，最大0.6x0.4cm。

双颈部、左锁骨上未见异常肿大淋巴结。

双侧颈内静脉管壁平整，管腔内清晰，未见明显栓子，CDFI:血流充盈良好。

English translation version (not used in this study):

The liver exhibits normal morphology and size with slightly heterogeneous echotexture, and no obvious space-occupying lesions are observed.

The biliary system is not dilated.

No significant space-occupying lesions are seen in the pancreas, bilateral kidneys, or adrenal regions.

The spleen is not enlarged.

No obvious enlarged lymph nodes are noted around the abdominal aorta.

Multiple small hypoechoic lymph nodes are observed in the right supraclavicular region, with the largest measuring 0.6 x 0.4 cm.

No abnormal enlarged lymph nodes are seen in the bilateral neck or left supraclavicular region.

The walls of the bilateral internal jugular veins are smooth, and the lumens are clear with no obvious thrombi. CDFI (Color Doppler Flow Imaging) shows good blood flow filling.

*Ground truth impressions of the example US report:*

腹部扫查未见明显占位

右锁骨上多发小淋巴结，观察

English translation version (not used in this study):

No obvious space-occupying lesions are detected in the abdominal scan.

Multiple small lymph nodes are observed in the right supraclavicular region, and follow-up is recommended.

*Impressions generated by Claude using zero-shot prompt:*

1. 肝脏回声欠均匀，考虑可能为脂肪肝或慢性肝病变。

2. 右锁骨上区域发现多发小淋巴结，最大径0.6cm，需进一步随访观察。

3. 腹部其他器官（胆系、胰腺、脾脏、双肾及肾上腺）未见明显异常。

4. 未见腹主动脉周围淋巴结肿大。

5. 双侧颈内静脉未见血栓形成。

English translation version (not used in this study):

1. The liver shows slightly heterogeneous echotexture, possibly suggesting fatty liver or chronic liver disease.

2. Multiple small lymph nodes are detected in the right supraclavicular region, with the largest diameter measuring 0.6 cm. Further follow-up and observation are recommended.

3. No significant abnormalities are observed in other abdominal organs (biliary system, pancreas, spleen, bilateral kidneys, and adrenal glands).

4. No enlarged lymph nodes are noted around the abdominal aorta.

5. No thrombosis is observed in the bilateral internal jugular veins.

*Impressions generated by Claude using one-shot prompt:*

1. 肝脏回声欠均匀，考虑肝实质病变

2. 右锁骨上多发小淋巴结，建议随访

English translation version (not used in this study):

1. The liver shows slightly heterogeneous echotexture, suggesting possible parenchymal liver disease.

2. Multiple small lymph nodes are observed in the right supraclavicular region, and follow-up is recommended.

*Impressions generated by Claude using three-shot prompt:*

1. 腹部扫查未见明显异常

2. 右锁骨上多发小淋巴结，建议随访

English translation version (not used in this study):

1. No obvious abnormalities are detected in the abdominal scan.

2. Multiple small lymph nodes are observed in the right supraclavicular region, and follow-up is recommended.

Table S1. Clinician I’s semantic evaluation results of the generated CT impressions.

| Metric | Prompt type | Tongyi Qianwen | ERNIE Bot | ChatGPT | Bard | Claude |
| --- | --- | --- | --- | --- | --- | --- |
| Completeness | Zero-shot | 4.90 | 4.83 | 4.80 | 4.87 | 4.59 |
|  | One-shot | 4.84 | 4.66 | 4.79 | 4.77 | 4.63 |
|  | Three-shot | 4.85 | 4.75 | 4.77 | 4.70 | 4.63 |
| Correctness | Zero-shot | 4.57 | 4.61 | 4.41 | 4.53 | 3.89 |
|  | One-shot | 4.52 | 4.54 | 4.44 | 4.55 | 4.06 |
|  | Three-shot | 4.64 | 4.54 | 4.45 | 4.44 | 4.03 |
| Conciseness | Zero-shot | 1.32 | 2.24 | 1.90 | 1.90 | 2.21 |
|  | One-shot | 2.87 | 4.38 | 2.56 | 3.59 | 2.96 |
|  | Three-shot | 2.95 | 3.99 | 3.10 | 3.81 | 3.16 |
| Verisimilitude | Zero-shot | 1.96 | 2.47 | 2.01 | 2.22 | 2.35 |
|  | One-shot | 3.21 | 4.03 | 2.66 | 3.36 | 3.09 |
|  | Three-shot | 3.15 | 3.81 | 3.13 | 3.52 | 3.24 |
| Replaceability | Zero-shot | 2.23 | 2.14 | 1.84 | 2.04 | 2.27 |
|  | One-shot | 2.64 | 2.91 | 2.16 | 2.31 | 2.80 |
|  | Three-shot | 2.63 | 2.58 | 2.26 | 2.39 | 2.90 |

Table S2. Clinician II’s semantic evaluation results of the generated CT impressions.

| Metric | Prompt type | Tongyi Qianwen | ERNIE Bot | ChatGPT | Bard | Claude |
| --- | --- | --- | --- | --- | --- | --- |
| Completeness | Zero-shot | 4.51 | 4.36 | 4.38 | 4.35 | 4.07 |
|  | One-shot | 4.40 | 4.30 | 4.41 | 4.24 | 4.10 |
|  | Three-shot | 4.50 | 4.45 | 4.42 | 4.22 | 4.13 |
| Correctness | Zero-shot | 3.77 | 3.62 | 4.03 | 3.27 | 3.43 |
|  | One-shot | 4.01 | 4.12 | 4.12 | 3.73 | 3.69 |
|  | Three-shot | 4.08 | 4.12 | 4.17 | 3.73 | 3.80 |
| Conciseness | Zero-shot | 1.35 | 2.49 | 2.57 | 2.29 | 2.26 |
|  | One-shot | 2.93 | 4.44 | 3.36 | 3.74 | 3.15 |
|  | Three-shot | 3.30 | 4.31 | 3.77 | 4.02 | 3.35 |
| Verisimilitude | Zero-shot | 2.22 | 2.74 | 2.88 | 2.49 | 2.50 |
|  | One-shot | 3.18 | 3.85 | 3.42 | 3.11 | 3.07 |
|  | Three-shot | 3.43 | 3.89 | 3.65 | 3.45 | 3.33 |
| Replaceability | Zero-shot | 1.83 | 2.72 | 2.94 | 2.45 | 2.61 |
|  | One-shot | 3.14 | 3.99 | 3.44 | 3.27 | 3.27 |
|  | Three-shot | 3.46 | 3.87 | 3.82 | 3.56 | 3.50 |

Table S3. Clinician III’s semantic evaluation results of the generated CT impressions.

| Metric | Prompt type | Tongyi Qianwen | ERNIE Bot | ChatGPT | Bard | Claude |
| --- | --- | --- | --- | --- | --- | --- |
| Completeness | Zero-shot | 4.98 | 4.99 | 4.87 | 4.99 | 5.00 |
|  | One-shot | 4.95 | 4.81 | 4.90 | 4.95 | 5.00 |
|  | Three-shot | 4.95 | 4.89 | 4.92 | 4.85 | 4.99 |
| Correctness | Zero-shot | 4.40 | 3.97 | 3.01 | 3.73 | 4.20 |
|  | One-shot | 4.24 | 4.07 | 3.83 | 3.87 | 4.13 |
|  | Three-shot | 4.27 | 4.08 | 3.75 | 3.99 | 4.10 |
| Conciseness | Zero-shot | 1.57 | 2.66 | 2.18 | 2.29 | 3.00 |
|  | One-shot | 3.09 | 4.66 | 3.13 | 3.82 | 3.49 |
|  | Three-shot | 3.32 | 4.47 | 3.79 | 4.31 | 3.50 |
| Verisimilitude | Zero-shot | 3.17 | 3.40 | 2.51 | 2.78 | 3.74 |
|  | One-shot | 3.77 | 4.44 | 3.54 | 3.91 | 4.04 |
|  | Three-shot | 3.89 | 4.35 | 3.93 | 4.24 | 4.11 |
| Replaceability | Zero-shot | 3.35 | 3.22 | 2.44 | 2.89 | 3.80 |
|  | One-shot | 3.61 | 3.72 | 3.21 | 3.40 | 3.99 |
|  | Three-shot | 3.58 | 3.74 | 3.24 | 3.54 | 4.08 |

Table S4. Clinician I’s semantic evaluation results of the generated PET-CT impressions.

| Metric | Prompt type | Tongyi Qianwen | ERNIE Bot | ChatGPT | Bard | Claude |
| --- | --- | --- | --- | --- | --- | --- |
| Completeness | Zero-shot | 3.81 | 3.94 | 4.07 | 3.86 | 3.82 |
|  | One-shot | 3.43 | - | 3.81 | 3.88 | 3.94 |
|  | Three-shot | 3.49 | - | 3.74 | 3.74 | 3.95 |
| Correctness | Zero-shot | 3.70 | 3.64 | 3.79 | 3.68 | 3.14 |
|  | One-shot | 3.61 | - | 3.64 | 3.68 | 3.06 |
|  | Three-shot | 3.66 | - | 3.56 | 3.57 | 3.05 |
| Conciseness | Zero-shot | 2.87 | 1.76 | 1.37 | 2.73 | 2.34 |
|  | One-shot | 3.59 | - | 1.47 | 2.20 | 1.70 |
|  | Three-shot | 3.75 | - | 1.98 | 2.56 | 1.67 |
| Verisimilitude | Zero-shot | 2.97 | 2.22 | 1.42 | 2.22 | 2.41 |
|  | One-shot | 3.41 | - | 1.94 | 2.33 | 2.12 |
|  | Three-shot | 3.46 | - | 2.33 | 2.63 | 2.02 |
| Replaceability | Zero-shot | 1.97 | 1.63 | 1.24 | 1.67 | 2.10 |
|  | One-shot | 1.99 | - | 1.60 | 1.58 | 1.96 |
|  | Three-shot | 1.93 | - | 1.70 | 1.66 | 1.92 |

Table S5. Clinician II’s semantic evaluation results of the generated PET-CT impressions.

| Metric | Prompt type | Tongyi Qianwen | ERNIE Bot | ChatGPT | Bard | Claude |
| --- | --- | --- | --- | --- | --- | --- |
| Completeness | Zero-shot | 3.73 | 3.87 | 3.89 | 3.49 | 3.83 |
|  | One-shot | 3.40 | - | 3.94 | 3.66 | 3.96 |
|  | Three-shot | 3.18 | - | 3.81 | 3.61 | 3.99 |
| Correctness | Zero-shot | 3.49 | 3.20 | 3.34 | 2.88 | 3.16 |
|  | One-shot | 3.23 | - | 3.17 | 2.68 | 3.24 |
|  | Three-shot | 3.03 | - | 3.21 | 2.89 | 3.29 |
| Conciseness | Zero-shot | 3.61 | 2.22 | 1.92 | 3.57 | 2.52 |
|  | One-shot | 3.97 | - | 2.02 | 3.02 | 2.05 |
|  | Three-shot | 4.09 | - | 2.69 | 3.20 | 2.11 |
| Verisimilitude | Zero-shot | 2.96 | 2.32 | 2.06 | 2.44 | 2.50 |
|  | One-shot | 2.77 | - | 2.16 | 2.31 | 2.29 |
|  | Three-shot | 2.66 | - | 2.48 | 2.46 | 2.31 |
| Replaceability | Zero-shot | 3.02 | 2.32 | 2.08 | 2.54 | 2.63 |
|  | One-shot | 2.77 | - | 2.21 | 2.25 | 2.44 |
|  | Three-shot | 2.56 | - | 2.54 | 2.48 | 2.60 |

Table S6. Clinician III’s semantic evaluation results of the generated PET-CT impressions.

| Metric | Prompt type | Tongyi Qianwen | ERNIE Bot | ChatGPT | Bard | Claude |
| --- | --- | --- | --- | --- | --- | --- |
| Completeness | Zero-shot | 4.07 | 4.01 | 3.99 | 3.88 | 4.93 |
|  | One-shot | 3.77 | - | 4.02 | 4.00 | 5.00 |
|  | Three-shot | 3.88 | - | 4.04 | 4.00 | 5.00 |
| Correctness | Zero-shot | 4.01 | 3.86 | 2.58 | 3.95 | 3.99 |
|  | One-shot | 3.92 | - | 3.84 | 4.00 | 4.00 |
|  | Three-shot | 3.96 | - | 3.84 | 3.96 | 4.00 |
| Conciseness | Zero-shot | 4.25 | 2.73 | 2.34 | 3.46 | 3.18 |
|  | One-shot | 4.13 | - | 2.93 | 3.51 | 2.44 |
|  | Three-shot | 4.56 | - | 3.84 | 3.67 | 2.52 |
| Verisimilitude | Zero-shot | 4.03 | 2.96 | 2.25 | 3.17 | 3.49 |
|  | One-shot | 3.96 | - | 3.26 | 3.45 | 3.15 |
|  | Three-shot | 4.06 | - | 3.85 | 3.51 | 2.99 |
| Replaceability | Zero-shot | 3.98 | 2.81 | 2.32 | 3.17 | 3.43 |
|  | One-shot | 3.89 | - | 3.25 | 3.42 | 3.15 |
|  | Three-shot | 3.82 | - | 3.73 | 3.47 | 2.94 |

Table S7. Clinician I’s semantic evaluation results of the generated Ultrasound impressions.

| Metric | Prompt type | Tongyi Qianwen | ERNIE Bot | ChatGPT | Bard | Claude |
| --- | --- | --- | --- | --- | --- | --- |
| Completeness | Zero-shot | 4.11 | 4.01 | 3.82 | 3.88 | 4.16 |
|  | One-shot | 4.10 | 4.01 | 3.67 | 3.75 | 4.19 |
|  | Three-shot | 4.20 | 4.08 | 3.95 | 3.71 | 4.34 |
| Correctness | Zero-shot | 3.57 | 3.53 | 3.56 | 3.35 | 3.19 |
|  | One-shot | 3.66 | 3.77 | 3.47 | 3.49 | 3.60 |
|  | Three-shot | 3.71 | 3.88 | 3.63 | 3.57 | 3.64 |
| Conciseness | Zero-shot | 1.05 | 1.77 | 1.38 | 1.69 | 1.77 |
|  | One-shot | 2.76 | 2.85 | 2.80 | 2.91 | 3.76 |
|  | Three-shot | 3.00 | 3.09 | 3.09 | 2.98 | 3.24 |
| Verisimilitude | Zero-shot | 1.41 | 2.09 | 1.69 | 1.97 | 2.07 |
|  | One-shot | 2.72 | 2.80 | 2.34 | 2.76 | 3.57 |
|  | Three-shot | 2.86 | 3.05 | 2.98 | 2.83 | 3.41 |
| Replaceability | Zero-shot | 1.66 | 1.80 | 1.77 | 1.32 | 2.04 |
|  | One-shot | 2.23 | 2.21 | 2.10 | 1.84 | 2.87 |
|  | Three-shot | 2.34 | 2.57 | 2.42 | 1.98 | 2.97 |

Table S8. Clinician II’s semantic evaluation results of the generated Ultrasound impressions.

| Metric | Prompt type | Tongyi Qianwen | ERNIE Bot | ChatGPT | Bard | Claude |
| --- | --- | --- | --- | --- | --- | --- |
| Completeness | Zero-shot | 4.36 | 4.32 | 4.17 | 3.96 | 3.92 |
|  | One-shot | 4.22 | 4.01 | 3.75 | 3.64 | 3.40 |
|  | Three-shot | 4.37 | 4.29 | 4.16 | 3.73 | 4.12 |
| Correctness | Zero-shot | 4.03 | 3.85 | 3.75 | 2.54 | 3.31 |
|  | One-shot | 3.96 | 3.88 | 3.77 | 2.96 | 3.39 |
|  | Three-shot | 4.04 | 4.14 | 3.95 | 3.31 | 3.93 |
| Conciseness | Zero-shot | 1.09 | 2.40 | 2.02 | 2.36 | 2.15 |
|  | One-shot | 3.21 | 3.44 | 3.56 | 3.67 | 3.90 |
|  | Three-shot | 3.58 | 3.81 | 3.88 | 3.77 | 3.59 |
| Verisimilitude | Zero-shot | 1.85 | 2.57 | 2.34 | 1.86 | 2.47 |
|  | One-shot | 3.14 | 3.13 | 3.13 | 2.62 | 3.19 |
|  | Three-shot | 3.54 | 3.71 | 3.51 | 2.92 | 3.63 |
| Replaceability | Zero-shot | 1.33 | 2.54 | 2.29 | 1.75 | 2.50 |
|  | One-shot | 3.13 | 3.25 | 3.11 | 2.57 | 3.20 |
|  | Three-shot | 3.67 | 3.82 | 3.59 | 2.93 | 3.75 |

Table S9. Clinician III’s semantic evaluation results of the generated Ultrasound impressions.

| Metric | Prompt type | Tongyi Qianwen | ERNIE Bot | ChatGPT | Bard | Claude |
| --- | --- | --- | --- | --- | --- | --- |
| Completeness | Zero-shot | 5.00 | 5.00 | 4.99 | 4.99 | 4.99 |
|  | One-shot | 4.99 | 5.00 | 5.00 | 4.98 | 5.00 |
|  | Three-shot | 4.96 | 5.00 | 5.00 | 4.97 | 5.00 |
| Correctness | Zero-shot | 4.40 | 4.08 | 4.13 | 4.15 | 4.23 |
|  | One-shot | 4.45 | 4.50 | 4.32 | 4.29 | 4.09 |
|  | Three-shot | 4.37 | 4.47 | 4.66 | 4.74 | 4.44 |
| Conciseness | Zero-shot | 2.00 | 3.13 | 3.10 | 3.22 | 2.32 |
|  | One-shot | 4.08 | 4.17 | 4.14 | 4.24 | 4.97 |
|  | Three-shot | 4.37 | 4.39 | 4.62 | 4.36 | 4.44 |
| Verisimilitude | Zero-shot | 3.00 | 4.01 | 4.06 | 4.06 | 3.17 |
|  | One-shot | 4.31 | 4.37 | 4.40 | 4.41 | 4.96 |
|  | Three-shot | 4.42 | 4.45 | 4.64 | 4.65 | 4.99 |
| Replaceability | Zero-shot | 3.83 | 4.01 | 4.05 | 4.04 | 2.95 |
|  | One-shot | 4.11 | 4.33 | 4.22 | 4.16 | 4.02 |
|  | Three-shot | 4.26 | 4.45 | 4.56 | 4.47 | 4.41 |


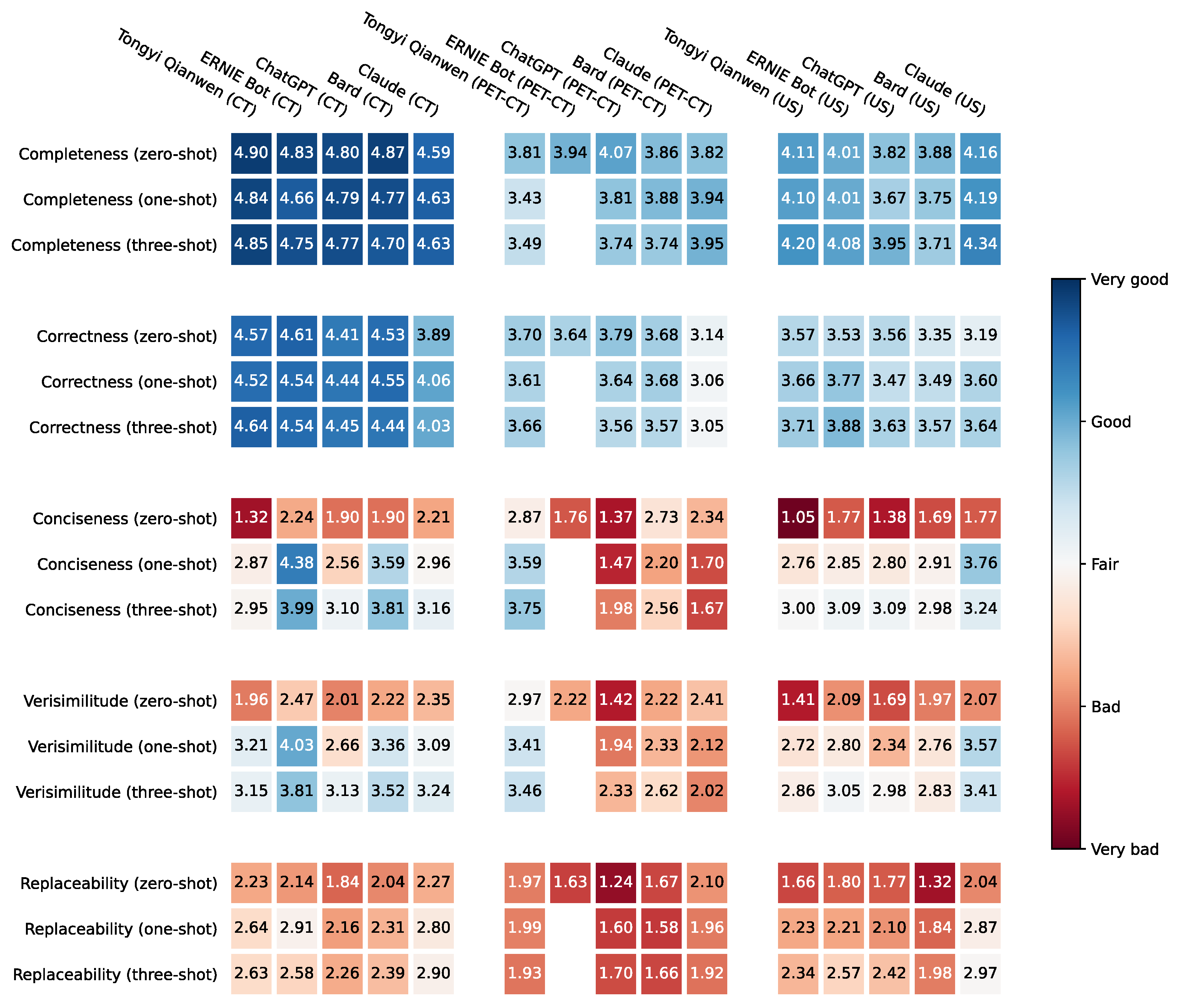


Figure S1. Heatmap visualization of clinician I’s human semantic evaluation results.


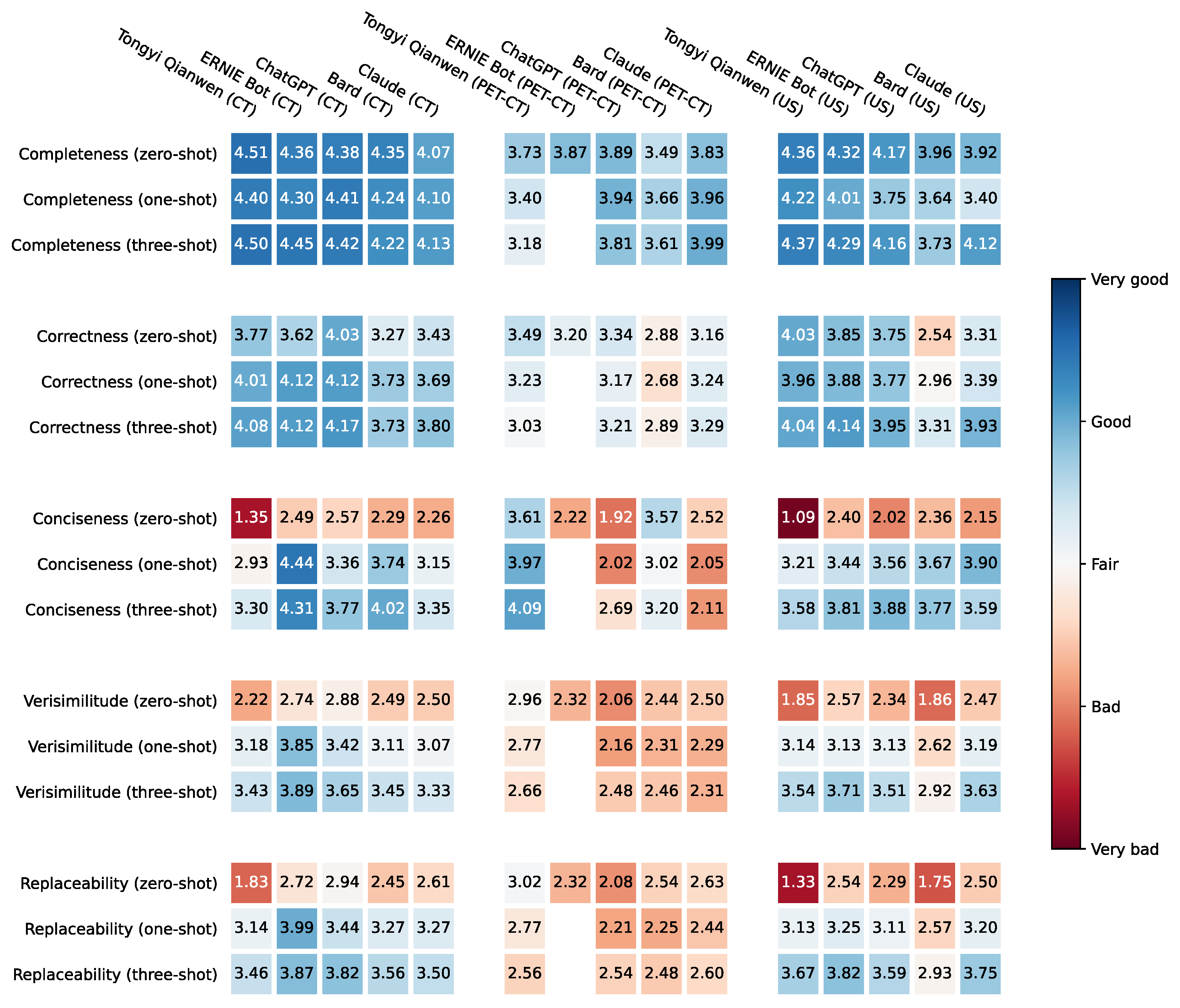


Figure S2. Heatmap visualization of clinician II’ s human semantic evaluation results.


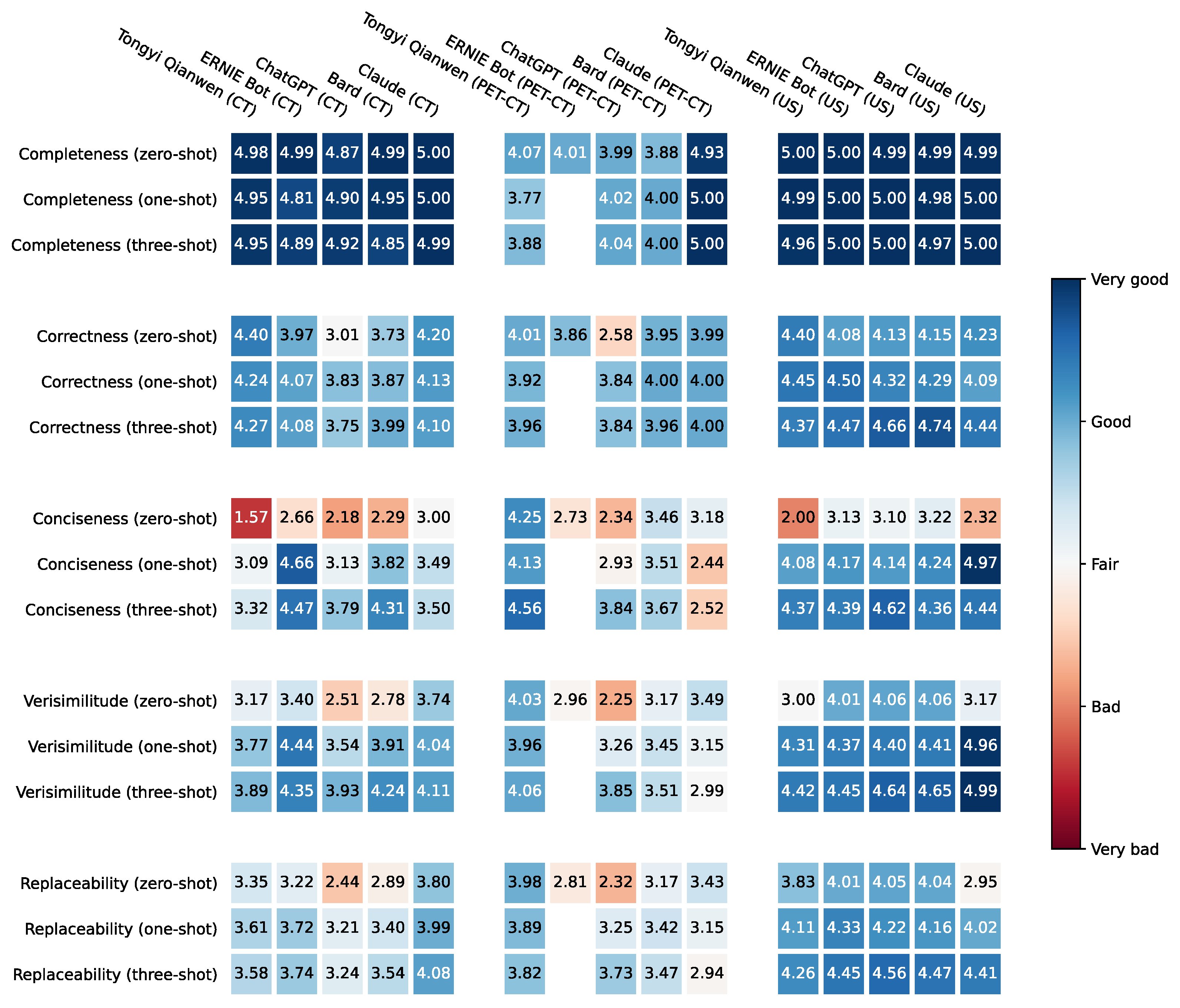


Figure S3. Heatmap visualization of clinician III’ s human semantic evaluation results.





Figure S4. Statistical test results of Clinician I’ s CT evaluation.





Figure S5. Statistical test results of Clinician II’ s CT evaluation.





Figure S6. Statistical test results of Clinician III’ s CT evaluation.





Figure S7. Statistical test results of Clinician I’ s PET-CT evaluation.





Figure S8. Statistical test results of Clinician II’ s PET-CT evaluation.





Figure S9. Statistical test results of Clinician III’ s PET-CT evaluation.





Figure S10. Statistical test results of Clinician I’ s Ultrasound evaluation.





Figure S11. Statistical test results of Clinician II’ s Ultrasound evaluation.





Figure S12. Statistical test results of Clinician III’ s Ultrasound evaluation.

Table S10. Automatic quantitative evaluation results of the generated raw CT impressions.

| Prompt type | Model | BLEU1 | BLEU2 | BLEU3 | BLEU4 | ROUGE-L | METEOR |
| --- | --- | --- | --- | --- | --- | --- | --- |
| Zero-shot | Tongyi Qianwen | 0.017 | 0.014 | 0.011 | 0.009 | 0.205 | 0.147 |
|  | ERNIE Bot | 0.089 | 0.073 | 0.061 | 0.052 | 0.291 | 0.234 |
|  | ChatGPT | 0.028 | 0.021 | 0.017 | 0.014 | 0.203 | 0.150 |
|  | Bard | 0.000 | 0.000 | 0.000 | 0.000 | 0.112 | 0.070 |
|  | Claude | 0.040 | 0.030 | 0.024 | 0.019 | 0.203 | 0.160 |
|  | Baichuan | 0.030 | 0.023 | 0.018 | 0.015 | 0.195 | 0.138 |
|  | ChatGLM | 0.009 | 0.007 | 0.005 | 0.004 | 0.174 | 0.125 |
|  | HuatuoGPT | 0.009 | 0.007 | 0.005 | 0.004 | 0.169 | 0.128 |
|  | ChatGLM-Med | 0.171 | 0.116 | 0.084 | 0.064 | 0.154 | 0.156 |
| One-shot | Tongyi Qianwen | 0.171 | 0.139 | 0.115 | 0.096 | 0.320 | 0.259 |
|  | ERNIE Bot | **0.431** | **0.356** | **0.302** | **0.257** | **0.463** | **0.458** |
|  | ChatGPT | 0.118 | 0.094 | 0.077 | 0.063 | 0.292 | 0.244 |
|  | Bard | 0.001 | 0.000 | 0.000 | 0.000 | 0.133 | 0.086 |
|  | Claude | 0.077 | 0.060 | 0.048 | 0.039 | 0.260 | 0.211 |
|  | Baichuan | 0.014 | 0.011 | 0.009 | 0.007 | 0.181 | 0.135 |
|  | ChatGLM | 0.042 | 0.033 | 0.027 | 0.022 | 0.223 | 0.181 |
|  | HuatuoGPT | 0.004 | 0.003 | 0.002 | 0.002 | 0.195 | 0.157 |
|  | ChatGLM-Med | 0.189 | 0.132 | 0.098 | 0.076 | 0.167 | 0.166 |
| Three-shot | Tongyi Qianwen | 0.224 | 0.183 | 0.153 | 0.129 | 0.350 | 0.299 |
|  | ERNIE Bot | 0.310 | 0.257 | 0.217 | 0.184 | 0.429 | 0.402 |
|  | ChatGPT | 0.166 | 0.136 | 0.114 | 0.096 | 0.337 | 0.298 |
|  | Bard | 0.001 | 0.001 | 0.000 | 0.000 | 0.137 | 0.090 |
|  | Claude | 0.078 | 0.063 | 0.052 | 0.043 | 0.284 | 0.230 |
|  | Baichuan | 0.115 | 0.091 | 0.075 | 0.062 | 0.293 | 0.256 |
|  | ChatGLM | 0.045 | 0.035 | 0.029 | 0.023 | 0.225 | 0.178 |
|  | HuatuoGPT | 0.000 | 0.000 | 0.000 | 0.000 | 0.148 | 0.108 |
|  | ChatGLM-Med | 0.150 | 0.095 | 0.063 | 0.045 | 0.140 | 0.140 |

Table S11. Automatic quantitative evaluation results of the generated raw PET-CT impressions.

| Prompt type | Model | BLEU1 | BLEU2 | BLEU3 | BLEU4 | ROUGE-L | METEOR |
| --- | --- | --- | --- | --- | --- | --- | --- |
| Zero-shot | Tongyi Qianwen | 0.438 | 0.335 | 0.264 | 0.212 | 0.316 | 0.382 |
|  | ERNIE Bot | 0.318 | 0.247 | 0.201 | 0.167 | 0.302 | 0.326 |
|  | ChatGPT | 0.159 | 0.119 | 0.093 | 0.074 | 0.226 | 0.227 |
|  | Bard | 0.113 | 0.082 | 0.064 | 0.051 | 0.210 | 0.197 |
|  | Claude | 0.325 | 0.247 | 0.191 | 0.151 | 0.288 | 0.329 |
|  | Baichuan | 0.100 | 0.076 | 0.060 | 0.048 | 0.216 | 0.212 |
|  | ChatGLM | 0.103 | 0.077 | 0.061 | 0.049 | 0.208 | 0.206 |
|  | HuatuoGPT | 0.110 | 0.082 | 0.066 | 0.054 | 0.203 | 0.193 |
|  | ChatGLM-Med | 0.090 | 0.059 | 0.044 | 0.035 | 0.094 | 0.145 |
| One-shot | Tongyi Qianwen | **0.476** | **0.368** | **0.294** | **0.240** | 0.340 | 0.424 |
|  | ERNIE Bot | - | - | - | - | - | - |
|  | ChatGPT | 0.221 | 0.166 | 0.129 | 0.102 | 0.248 | 0.266 |
|  | Bard | 0.063 | 0.048 | 0.038 | 0.031 | 0.196 | 0.174 |
|  | Claude | 0.339 | 0.269 | 0.216 | 0.176 | 0.323 | 0.358 |
|  | Baichuan | 0.066 | 0.051 | 0.041 | 0.034 | 0.210 | 0.197 |
|  | ChatGLM | 0.199 | 0.149 | 0.117 | 0.094 | 0.232 | 0.246 |
|  | HuatuoGPT | 0.083 | 0.062 | 0.048 | 0.039 | 0.188 | 0.172 |
|  | ChatGLM-Med | - | - | - | - | - | - |
| Three-shot | Tongyi Qianwen | 0.439 | 0.340 | 0.272 | 0.221 | 0.354 | **0.450** |
|  | ERNIE Bot | - | - | - | - | - | - |
|  | ChatGPT | 0.309 | 0.234 | 0.183 | 0.147 | 0.274 | 0.309 |
|  | Bard | 0.105 | 0.080 | 0.064 | 0.052 | 0.217 | 0.204 |
|  | Claude | 0.362 | 0.297 | 0.246 | 0.207 | **0.356** | 0.394 |
|  | Baichuan | - | - | - | - | - | - |
|  | ChatGLM | 0.188 | 0.143 | 0.115 | 0.094 | 0.215 | 0.227 |
|  | HuatuoGPT | - | - | - | - | - | - |
|  | ChatGLM-Med | - | - | - | - | - | - |

Table S12. Automatic quantitative evaluation results of the generated raw Ultrasound impressions.

| Prompt type | Model | BLEU1 | BLEU2 | BLEU3 | BLEU4 | ROUGE-L | METEOR |
| --- | --- | --- | --- | --- | --- | --- | --- |
| Zero-shot | Tongyi Qianwen | 0.000 | 0.000 | 0.000 | 0.000 | 0.122 | 0.083 |
|  | ERNIE Bot | 0.014 | 0.013 | 0.011 | 0.010 | 0.252 | 0.187 |
|  | ChatGPT | 0.001 | 0.001 | 0.001 | 0.001 | 0.157 | 0.105 |
|  | Bard | 0.000 | 0.000 | 0.000 | 0.000 | 0.090 | 0.053 |
|  | Claude | 0.009 | 0.007 | 0.005 | 0.004 | 0.185 | 0.136 |
|  | Baichuan | 0.006 | 0.006 | 0.005 | 0.004 | 0.231 | 0.167 |
|  | ChatGLM | 0.001 | 0.001 | 0.001 | 0.000 | 0.163 | 0.109 |
|  | HuatuoGPT | 0.000 | 0.000 | 0.000 | 0.000 | 0.118 | 0.073 |
|  | ChatGLM-Med | 0.165 | 0.131 | 0.109 | 0.093 | 0.162 | 0.138 |
| One-shot | Tongyi Qianwen | 0.122 | 0.101 | 0.082 | 0.066 | 0.319 | 0.294 |
|  | ERNIE Bot | 0.072 | 0.062 | 0.055 | 0.048 | 0.304 | 0.257 |
|  | ChatGPT | 0.008 | 0.007 | 0.006 | 0.006 | 0.239 | 0.193 |
|  | Bard | 0.000 | 0.000 | 0.000 | 0.000 | 0.106 | 0.065 |
|  | Claude | 0.022 | 0.018 | 0.014 | 0.012 | 0.205 | 0.151 |
|  | Baichuan | 0.049 | 0.042 | 0.036 | 0.032 | 0.278 | 0.226 |
|  | ChatGLM | 0.005 | 0.004 | 0.003 | 0.003 | 0.190 | 0.137 |
|  | HuatuoGPT | 0.000 | 0.000 | 0.000 | 0.000 | 0.104 | 0.073 |
|  | ChatGLM-Med | **0.165** | 0.131 | 0.111 | 0.097 | 0.143 | 0.124 |
| Three-shot | Tongyi Qianwen | 0.150 | 0.131 | 0.115 | 0.100 | 0.408 | 0.365 |
|  | ERNIE Bot | 0.042 | 0.038 | 0.035 | 0.033 | 0.351 | 0.286 |
|  | ChatGPT | 0.151 | **0.137** | **0.126** | **0.115** | **0.441** | **0.396** |
|  | Bard | 0.000 | 0.000 | 0.000 | 0.000 | 0.111 | 0.068 |
|  | Claude | 0.094 | 0.086 | 0.078 | 0.070 | 0.359 | 0.289 |
|  | Baichuan | 0.098 | 0.081 | 0.069 | 0.059 | 0.270 | 0.224 |
|  | ChatGLM | 0.003 | 0.002 | 0.002 | 0.002 | 0.167 | 0.115 |
|  | HuatuoGPT | 0.000 | 0.000 | 0.000 | 0.000 | 0.096 | 0.062 |
|  | ChatGLM-Med | 0.131 | 0.107 | 0.093 | 0.083 | 0.141 | 0.118 |





Figure S13. Heatmap visualization of automatic quantitative assessment for the raw generated CT, PET-CT, and US impressions.


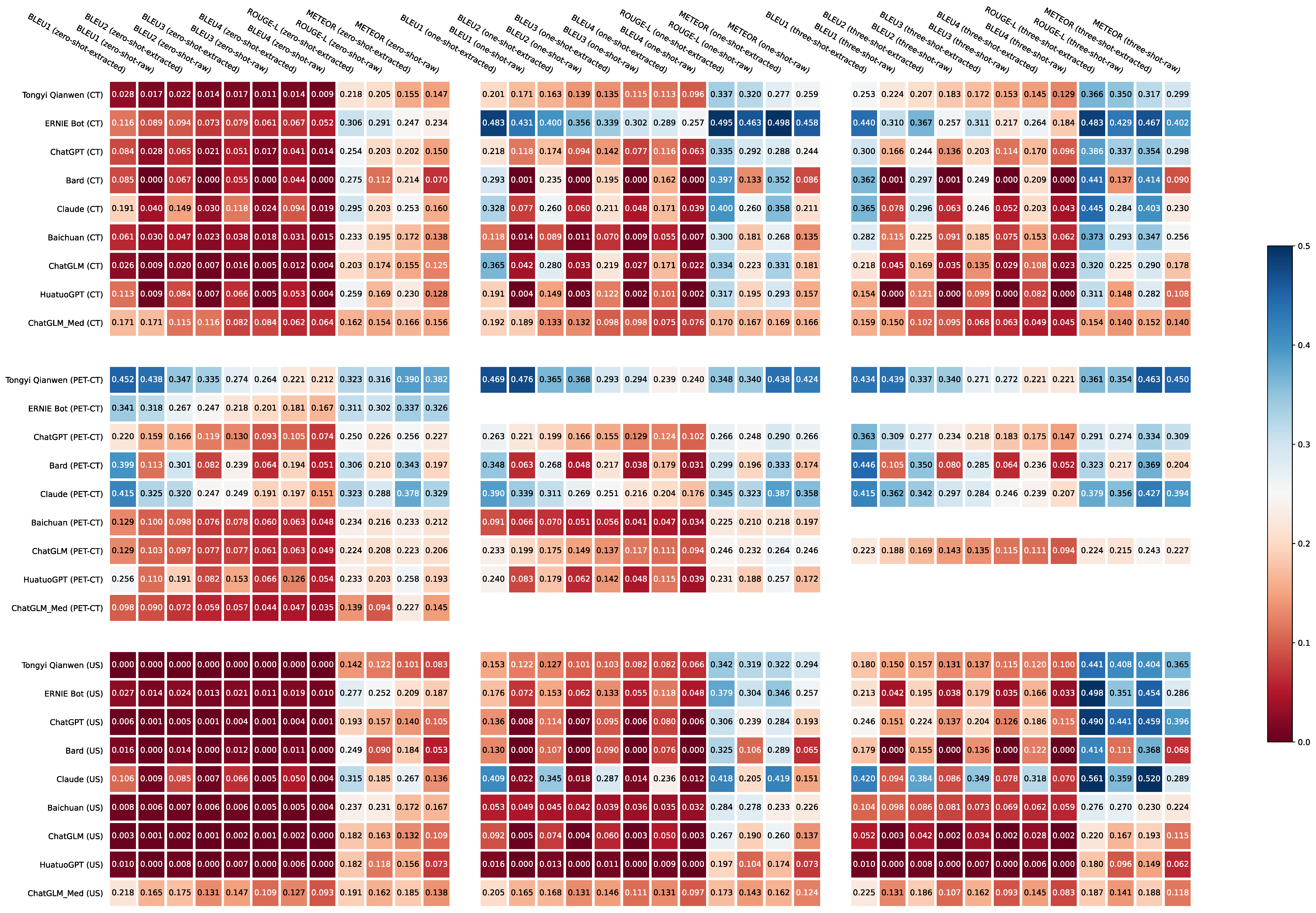


Figure S14. Comparison of automatic quantitative assessment of the raw and extracted generated impressions.
